# IER2-induced senescence drives melanoma invasion through osteopontin

**DOI:** 10.1038/s41388-021-02027-6

**Published:** 2021-10-05

**Authors:** Lenka Kyjacova, Rafael Saup, Kerstin Rönsch, Sabine Wallbaum, Stefanie Dukowic-Schulze, Amelia Foss, Sandra D. Scherer, Melanie Rothley, Antje Neeb, Nicole Grau, Wilko Thiele, Sonja Thaler, Natascha Cremers, Carsten Sticht, Norbert Gretz, Boyan K. Garvalov, Jochen Utikal, Jonathan P. Sleeman

**Affiliations:** 1 Department of Microvascular Biology and Pathobiology, European Center for Angioscience (ECAS), Medical Faculty Mannheim, University of Heidelberg, Mannheim, Germany; 2 Institute of Biological and Chemical Systems—Biological Information Processing (IBCS-BIP), Karlsruhe Institute of Technology (KIT) Campus Nord, Karlsruhe, Germany; 3 Medical Research Center, Medical Faculty Mannheim, University of Heidelberg, Mannheim, Germany; 4 Skin Cancer Unit, German Cancer Research Center (DKFZ), Heidelberg, Germany; 5 Department of Dermatology, Venereology and Allergology, University Medical Center Mannheim, University of Heidelberg, Mannheim, Germany; 6 Present Address: Eurofins GATC Biotech GmbH, Konstanz, Germany; 7 Present Address: Department of Radiation Oncology, Heidelberg University Hospital, Heidelberg, Germany

**Keywords:** Cytokines, Melanoma, Cell migration, Cell signalling, Senescence

## Abstract

Expression of the immediate-early response gene IER2 has been associated with the progression of several types of cancer, but its functional role is poorly understood. We found that increased IER2 expression in human melanoma is associated with shorter overall survival, and subsequently investigated the mechanisms through which IER2 exerts this effect. In experimental melanoma models, sustained expression of IER2 induced senescence in a subset of melanoma cells in a p53/MAPK/AKT-dependent manner. The senescent cells produced a characteristic secretome that included high levels of the extracellular phosphoglycoprotein osteopontin. Nuclear localization of the IER2 protein was critical for both the induction of senescence and osteopontin secretion. Osteopontin secreted by IER2-expressing senescent cells strongly stimulated the migration and invasion of non-senescent melanoma cells. Consistently, we observed coordinate expression of IER2, p53/p21, and osteopontin in primary human melanomas and metastases, highlighting the pathophysiological relevance of IER2-mediated senescence in melanoma progression. Together, our study reveals that sustained IER2 expression drives melanoma invasion and progression through stimulating osteopontin secretion via the stochastic induction of senescence.

## Introduction

The term “immediate-early response” (IER) describes the collective transient expression of immediate-early genes (IEGs) upon exposure to a variety of extracellular cues, including growth factors, mitogens, and stressors such as ultraviolet (UV) irradiation and toxins [1, 2]. Constitutive IEG expression, for example, caused by disturbed mitogen-activated protein kinase (MAPK) signaling that is often found in cancer cells, is associated with tumor progression and metastasis [3–5].

The immediate early response protein 2 (IER2, also called ETR101 and CHX1 in humans or Pip92 in the mouse; hereafter referred to as IER2, regardless of species) is a poorly investigated IEG that is rapidly but transiently induced in response to a variety of growth factors, and other mitogenic and differentiation-inducing stimuli [6–9]. The IER2 protein is short-lived due to multiple PEST sequences, contains a putative bipartite nuclear localization signal (NLS), and has limited homology to JunB and JunD [9, 10]. IER2 possesses no obvious DNA-binding motif but has been implicated in transcriptional regulation [11]. IER2 has also been suggested to regulate the activity of the protein phosphatase PP2A [12]. Cell density influences the subcellular localization of the IER2 protein, and the release of cells from contact inhibition results in translocation of IER2 from the cytoplasm to the nucleus [13]. During embryonic development, IER2 plays a role in determining left–right asymmetry [14].

We previously reported that IER2 is aberrantly and constitutively expressed in a wide variety of human tumors in comparison to the corresponding non-transformed tissue. Ectopic overexpression of IER2 in poorly metastatic pancreatic tumor cells promoted their motility and invasiveness in vitro and stimulated metastasis in vivo. Consistently, we found that IER2 is a predictor of poor metastasis-free and overall survival of colorectal adenocarcinoma patients [13]. More recently, others have demonstrated a critical role for IER2 in the regulation of hepatocellular carcinoma migration and invasion via an integrin β1-focal adhesion kinase (FAK)-Src-paxillin signaling pathway and Rho GTPases [15–17], and in the regulation of endothelial cell motility, cell–matrix adhesion and in vitro angiogenesis [18]. To date, however, the association between IER2 expression and tumor progression has not been explained mechanistically, in particular the consequence of the constitutive rather than transient expression that is observed in many cancers.

Here, we report that sustained expression of IER2 in fibroblasts stochastically induces cellular senescence. Senescence is typified by durable cell cycle arrest and an enlarged flattened morphology. The development of senescence involves proliferative arrest in response to sustained and robust activation of the p16^INK4a^–Rb (retinoblastoma) and/or p53–p21^WAF1/CIP1^ tumor suppressor pathways [19, 20]. Features of senescent cells include an enlarged morphology, positivity for senescence-associated β-galactosidase, increased ploidy, and the secretion of a cocktail of pro-inflammatory cytokines, growth factors and matrix remodeling enzymes termed the senescence-associated secretory phenotype (SASP) [21]. The specific content of the SASP depends on the cell type and the senescence inducer [22, 23].

In the context of cancer, cellular senescence can both restrict and promote tumorigenesis and progression. On the one hand, it represents a first-line barrier against neoplastic transformation and tumor initiation [19, 24, 25]. On the other hand, senescence can stimulate tumor growth and progression through cell-autonomous effects such as increasing invasive, tumorigenic, and cancer cell stemness properties [26, 27], as well as through non-cell-autonomous effects mediated by the SASP [28], including the recruitment of immature myeloid cells [29] and the promotion of metastasis [30].

In the present study, we report that IER2 expression can be constitutively increased in human melanomas and that this correlates with poor overall survival. By mimicking this constitutive expression in experimental models, we show that similar to the situation in fibroblasts, IER2 induces p53-dependent senescence in a fraction of melanoma cells and that this is associated with a characteristic SASP. A major component of the IER2-driven SASP is osteopontin (OPN), which plays a central role in driving melanoma cell invasion. These findings reveal an important role for IER2 in melanoma progression, highlight the role of senescence and the SASP in this process, and identify potential targets for therapeutic intervention.

## Results

### IER2 induces senescence in murine 3T3 fibroblasts

**Fig. 1:**
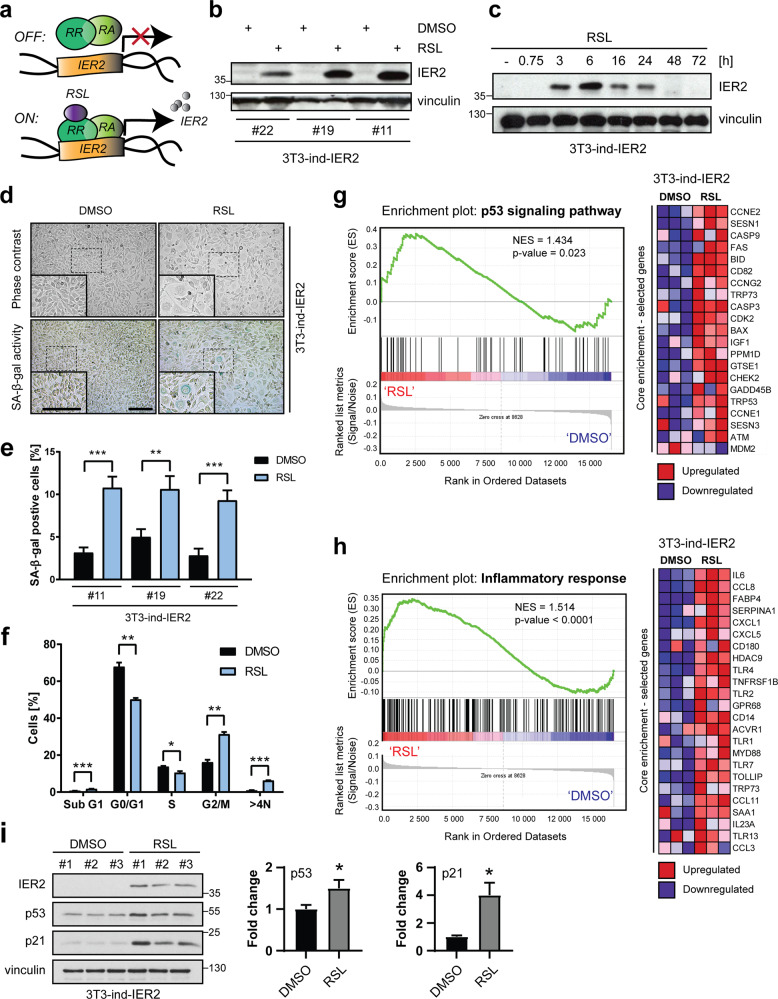
IER2 induces senescence in murine 3T3 fibroblasts. **a** Schematic representation of the RheoSwitch mammalian inducible expression system. RheoReceptor-1 (RR) and RheoActivator (RA) expressed from the pNEBR-R1 plasmid regulate transcription of mouse IER2 cloned into the pNEBR-X1 vector. **b** Western blot analysis of IER2 and vinculin (loading control) in three separate clones of NIH/3T3 mouse fibroblasts (3T3-ind-IER2 #11, #19, and #22), treated with DMSO or 0.2 μM RSL. **c** Western blot analysis of IER2 and vinculin in 3T3-ind-IER2 #11 cells treated with 0.2 μM RSL at the indicated time points. **d** Representative phase-contrast microscopic images (upper panel) and staining for SA-β-galactosidase (SA-β-gal) activity (blue, lower panel) of 3T3-ind-IER2 #11 cells treated for 3 days with DMSO or RSL (3 × 0.5 μM). Scale bars, 100 μm. **e** Percentage of SA-β-gal-positive 3T3-ind-IER2 #11, #19, and #22 cells treated either with DMSO or RSL (3 × 0.5 μM). Bars represent mean + SEM. More than 1000 cells per condition from 11 to 15 microscopic images were analyzed. **f** Cell cycle distribution of 3T3-ind-IER2 #11 cells measured by flow cytometry after treatment with DMSO or RSL (3 × 0.5 μM). Bars represent mean + SEM (*n* = 3; 30,000 cells per replicate were analyzed). **g**, **h** GSEA enrichment plots and corresponding heat maps of selected differentially expressed genes belonging to the “p53 signaling pathway” (using KEGG annotations, **g**) or “Inflammatory response” (using GOBP annotations, **h**) associated with elevated IER2 expression. The red and blue colors in the bar-code plot indicate over- and under-expression in the mRNA. NES normalized enrichment score. **i** Western blot analysis of IER2, p53, p21, and vinculin in 3T3-ind-IER2 #11 cells treated with either DMSO or RSL (3 × 0.5 μM). Bars represent vinculin-normalized background-corrected integrated signal densities + SEM from three biological replicates. Data are normalized to DMSO-treated cells. Statistical significance was determined by an unpaired Student’s *t* test. *p < 0.05, **p < 0.01, ***p < 0.001.

To investigate the impact of sustained IER2 expression on cell behavior, we engineered mouse 3T3 fibroblasts to inducibly express IER2 in response to RheoSwitch ligand 1 (RSL-1; hereafter referred to as RSL), a pharmacologically inert diacylhydrazine [31–33]. IER2 induction against very low basal levels was achieved after stimulation of the cells with RSL (Figs. S1a, 1a, 1b), which reached maximum expression at 6 h and diminished to basal levels within 48 h (Fig. 1c). To maintain IER2 expression over periods exceeding 24 h, cells were therefore treated daily with RSL (Fig. S1b). While sustained RSL-driven IER2 expression had no effect on viability (Fig. S1c), striking morphological changes and cell division abnormalities reminiscent of cellular senescence were observed in a subset of 3T3 cells (Video S1). Consistently, induction of IER2 expression resulted in increased senescence-associated β-galactosidase activity in a portion of the cells (Fig. 1d, e), which is a marker of cellular senescence [34]. Furthermore, IER2-expressing cells accumulated in the G2/M phase of the cell cycle and exhibited elevated ploidy (Fig. 1f), again indicative of the induction of senescence.

To understand the transcriptional response to continuous IER2 expression, we performed differential gene expression profiling of RSL-treated IER2-inducible 3T3 cells relative to controls. Gene set enrichment analysis (GSEA) [35] using Kyoto encyclopedia of genes and genomes (KEGG) and gene ontology biological processes annotations revealed that IER2-induced genes are involved in processes, including lipid metabolism, immune/inflammatory, cytokine production, and cell cycle regulation (Table S1). Importantly, the expression of TRP53 (mouse homolog of p53), the main regulator of senescence-associated cell cycle arrest [36], was increased, together with the p53 signaling pathway gene set. Correspondingly, expression of MDM2, a negative regulator of p53 [37] was decreased (Fig. 1g). In addition, several SASP-associated factors [28] including IL-6, CCL8, CXCL1, CXCL5, CCL3, and CCL11 were induced in response to IER2, together with SAA1, which has been reported to amplify the expression of SASP-related factors via the Toll-like receptors TLR2/TLR4 [38] (Fig. 1h). Western blotting confirmed increased expression of p53 in IER2-expressing 3T3 cells, as well as of the p53-target gene p21^waf1/cip1^ (hereafter p21) (Fig. 1i). In wild-type UV-irradiated 3T3 cells, IER2 induction preceded p53 activation (Fig. S1d) and was required for p53 stabilization (Fig. S1e).

Together, these findings demonstrate that constitutive IER2 expression stochastically promotes senescence in mouse fibroblasts, and point to a potential functional relationship between IER2 and p53.

### Constitutive IER2 expression correlates with poor prognosis in melanoma

**Fig. 2:**
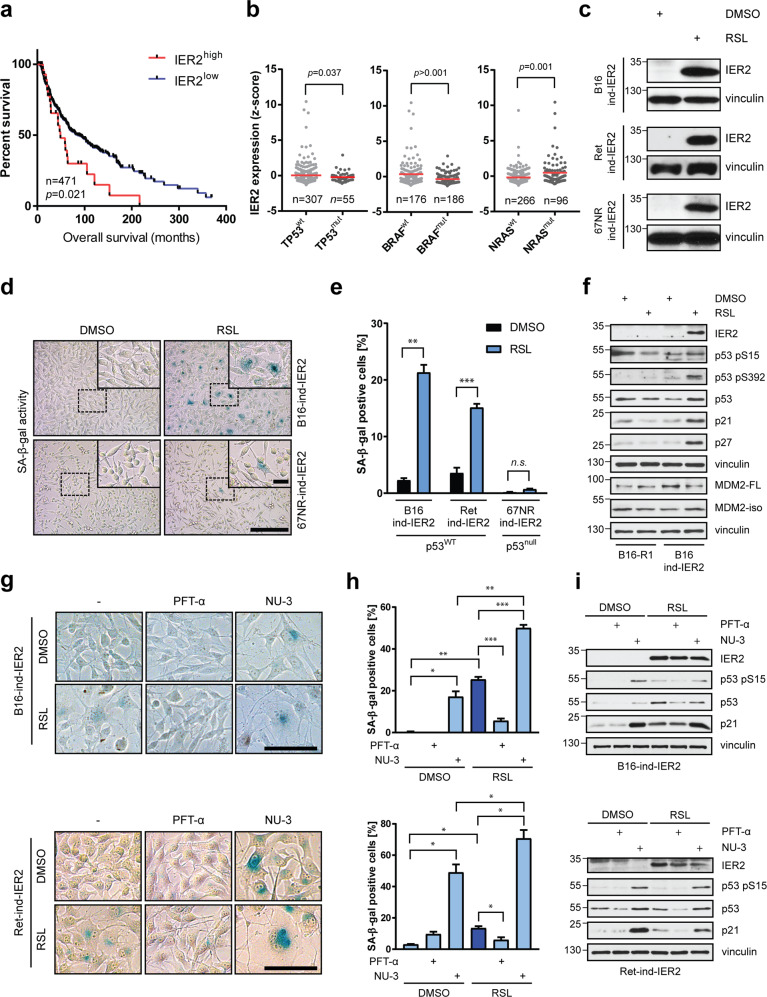
IER2 requires p53–p21 to promote senescence in murine melanoma cells. **a** Kaplan–Meier analysis of the overall survival of the TCGA skin cutaneous melanoma cohort (*n* = 471) stratified according to low (IER2^low^) and high (IER2^high^) IER2 expression. **b** IER2 expression in the skin cutaneous melanoma cohort from (**a**), with patients stratified according to p53 (left), BRAF (middle) and NRAS (right) mutational status. The red lines represent the means. **c** Western blot analysis of IER2 and vinculin (loading control) in B16-ind-IER2 #51, Ret-ind-IER2 #69, and 67NR-ind-IER2 #37 RheoSwitch cells treated either with DMSO or RSL (3 × 0.5–1.0 μM). **d** Staining for SA-β-galactosidase activity (blue) in B16-ind-IER2 #51 and 67NR-ind-IER2 #37 cells treated either with DMSO or with RSL (3 × 0.5 μM). Scale bar, 100 μm; inset 25 μm. **e** Percentage of SA-β-galactosidase-positive cells in B16-ind-IER2 #51, Ret-ind-IER2 #69, and 67NR-ind-IER2 #37 cells after stimulation with DMSO or RSL (3 × 0.5–1.0 μM; *n* = 3). **f** Western blot analysis of the indicated proteins in parental B16-R1 #2 and B16-ind-IER2 #51 cells after stimulation with either DMSO or RSL (3 × 0.5 μM). **g** Representative images of staining for SA-β-galactosidase activity (blue) and **h** quantification of SA-β-galactosidase staining in B16-ind-IER2 #51 cells (upper panels) and Ret-ind-IER2 #69 cells (bottom panels) treated with PFT-α (20 μM) or NU-3 (5 μM) with or without stimulation with RSL (3 × 0.5 μM); *n* = 3. Scale bars, 100 μm. **i** Western blot analysis of IER2, p53 pS15, p53, and p21 in B16-ind-IER2 #51 (upper panel) and Ret-ind-IER2 #69 cells (bottom panel) treated as in **g**. Bars represent mean + SEM; *n* = 3. Statistical significance was determined using an unpaired Student’s *t* test. n.s. nonsignificant (*p* > 0.05), *p < 0.05, **p < 0.01, ***p < 0.001.

Melanoma has high intrinsic potential for dissemination [39] and a high rate of metastatic spread compared to other cancers [40]. IER2 promotes metastasis and is associated with poor prognosis in colorectal cancer patients [13]. To investigate whether IER2 might play a similar role in human melanoma, we analyzed the TCGA skin cutaneous melanoma dataset. Kaplan–Meier analysis revealed that melanoma patients with high IER2 expression (Fig. S2a) had a significantly shorter overall survival compared to patients with lower IER2 levels (Fig. 2a). To analyze whether high IER2 expression is linked to common driver mutations in melanoma [41], tumors were stratified based on their *BRAF*, *NRAS*, and *TP53* mutation status. In line with the mutual exclusivity of *BRAF* and *NRAS* mutations in melanoma [42], we found that high IER2 expression is associated with wild-type *BRAF* and mutated *NRAS*. Importantly, high IER2 expression was almost exclusively associated with wild-type p53 status (Fig. 2b), consistent with our findings that p53 levels are increased in melanoma cells upon sustained IER2 expression (see below).

To determine whether IER2 expression correlates with the proliferative or the invasive potential of melanoma cells, we analyzed a dataset of human melanoma cell lines utilizing the Heuristic Online Phenotype Prediction algorithm (HOPP) [43–45]. This revealed significantly higher IER2 expression in melanoma cells that exhibited an intermediate or invasive phenotype (Fig. S2b, Table S2). Thus, similar to our previous work with pancreatic tumor cells [13], IER2 expression is associated with enhanced invasiveness of melanoma cells.

### IER2 stochastically induces senescence in melanoma cells via the p53–p21 axis

To investigate the impact of constitutive IER2 expression in melanoma, we generated IER2-inducible RheoSwitch B16–F10 (B16) and Ret mouse melanoma cells, which both harbor wild-type p53 (p53^WT^) and express only very low levels of IER2. To control for the effect of p53 status, in the absence of a suitable melanoma cell line we similarly created IER2-inducible 67NR mouse breast adenocarcinoma cells, which are p53-deficient (Fig. S2c) and likewise express very low endogenous levels of IER2 (Fig. 2c). To achieve sustained IER2 expression over periods exceeding 24 h, cells were treated with RSL daily (Fig. S2d). Similar to the case with fibroblasts, induction of IER2 expression in B16 and Ret cells led to the development of senescence in a proportion of the cells (Fig. 2d, e), as well as to the accumulation of cells in the G2/M phase of the cell cycle, and to a higher number of >4 N polyploid cells (Fig. S2e). By contrast, the p53-deficient (p53^null^) 67NR cells remained unaffected by IER2 overexpression (Fig. 2d, e). Importantly, ectopic re-introduction of wild-type p53 into two independent IER2-inducible 67NR clonal cell lines enhanced the ability of IER2 to induce senescence (Fig. S2f–h), indicating that wild-type p53 is required for the efficient induction of senescence by IER2.

In concordance with the results obtained with mouse 3T3 fibroblasts, ectopic expression of IER2 in B16 cells led to p53 stabilization and activation, as evidenced by phosphorylation of p53 on serine 15 and 392. Consistently, IER2 expression resulted in accumulation of the 48 kDa MDM2 isoform (MDM2-iso), an MDM2 splice variant that stabilizes/activates p53 through competitive binding to MDM2-full length (MDM2-FL), which inhibits the MDM2-FL–p53 interaction and thereby reduces p53 degradation [46–48]. Moreover, the p53 target gene and cell cycle regulator p21, together with p27^KIP1^ (p27) were also elevated upon IER2 induction (Fig. 2f), consistent with cell cycle arrest. Unchanged Rb (retinoblastoma) phosphorylation and protein levels, together with unaffected p16^INK4a^ (p16) levels indicate that IER2-mediated senescence is independent of the tumor-suppressor p16-Rb pathway [49] (Fig. S2i). RSL alone had no effect on cellular morphology, nor on the activation of the p53–p21 axis in control B16 cells, which were stably transfected with pNEBR-R1 plasmid alone (B16-R1; Figs. 2f and S2j). IER2 also had no impact on p53 mRNA levels, suggesting that IER2 regulates p53 post-transcriptionally (Fig. S3a, b). Consistently, p53 inhibition with pifithrin-α (PFT-α, Fig. 2g–i), as well as siRNA-mediated silencing of p53 (Fig. S2m, n) abrogated IER2-induced senescence, whereas stabilization of p53 by the MDM2 inhibitor nutlin-3 (NU-3) elicited a senescent phenotype in both B16 and Ret cells (Fig. 2g–i).

Next, we investigated whether IER2 is involved in p53 regulation following genotoxic stress. B16 cells were treated with the chemotherapy drugs doxorubicin and camptothecin with and without induction of IER2 expression. Elevated IER2 levels promoted earlier stabilization and activation of p53, as well as an earlier increase in p21 levels in response to both chemotherapeutics (Fig. S2k, l).

Together these data demonstrate that IER2 contributes to p53 regulation under both normal and stress conditions and that p53 is required for IER2-induced senescence.

### IER2 requires active AKT and MAPK signaling to promote p53-dependent senescence in mouse melanoma cells

**Fig. 3:**
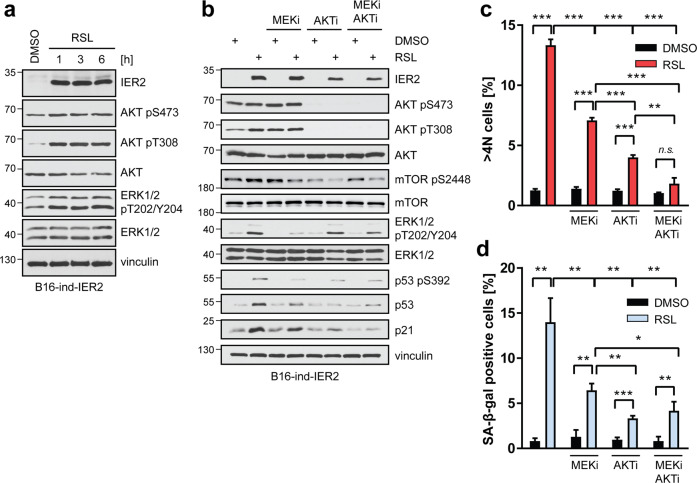
IER2 requires active AKT and ERK signaling to promote p53-dependent senescence in melanoma cells. **a** Western blot analysis of IER2, AKT phosphorylated on serine 473 (pS473) and threonine 308 (pT308), AKT, ERK phosphorylated on threonine 202/tyrosine 204 (pT202/Y204), ERK1/2, and vinculin (loading control) in B16-ind-IER2 #51 cells treated with DMSO or RSL (3 × 0.5 μM), harvested at indicated time points after the last dose of RSL. **b** Western blot analysis of the indicated proteins in B16-ind-IER2 #51 cells treated with MEKi (3 × 1 μM) and/or AKTi (3 × 1 μM), with or without stimulation with RSL (3 × 0.5 μM). **c** DRAQ5 labeling of nuclear DNA followed by flow cytometry quantification of >4 N B16-ind-IER2 #51 cells treated either with DMSO or with RSL (3 × 0.5 μM) in combination with MEKi (3 × 1 μM) and/or AKTi (3 × 1 μM). **d** Percentage of SA-β-galactosidase-positive B16-ind-IER2 #51 cells treated as in (**c**). Bars represent means + SEM; *n* = 3. Statistical significance was determined using an unpaired Student’s *t* test. n.s. nonsignificant (*p* > 0.05), *p < 0.05, **p < 0.01, ***p < 0.001.

The RAS–RAF–MEK–ERK (MAPK) and PI3K-AKT (AKT) signaling pathways are key players in melanoma progression [50]. Oncogenic mutations that hyperactivate ERK and AKT can promote p53-dependent oncogene-induced senescence in a cell context-dependent manner [49, 51]. Western blot analysis revealed rapid activation of both the MAPK and AKT pathways in response to IER2, as indicated by elevated phosphorylation of ERK1/2 at threonine 202 and tyrosine 204, and AKT at serine 473 and tyrosine 308 (Fig. 3a). These results suggest that IER2 may induce senescence via these pathways.

To investigate a possible role for the MAPK and AKT pathways in IER2-induced senescence, we inhibited the MAPK (MEKi) and AKT (AKTi) pathways alone and/or in combination in melanoma cells induced to express IER2, and compared p53 and p21 levels. MEK inhibition modestly decreased p53/p21 levels compared to untreated RSL-stimulated cells, while AKT inhibition completely abrogated IER2-induced p53 stabilization and substantially reduced p21 levels (Fig. 3b). Furthermore, both AKT and MEK inhibition abrogated the IER2-induced activation of mTOR (Fig. 3b), which is involved in the induction of senescence via p53 [51, 52]. Consistently, cells expressing IER2 showed a significant decrease in polyploidy (Figs. 3c, S3c, d) as well as in SA-β-galactosidase activity in the presence of MEKi and/or AKTi (Fig. S3e, Fig. 3d).

These results reveal that MAPK and AKT signaling is mechanistically linked to IER2-induced senescence in melanoma.

### Osteopontin is a major component of the IER2-induced SASP

**Fig. 4:**
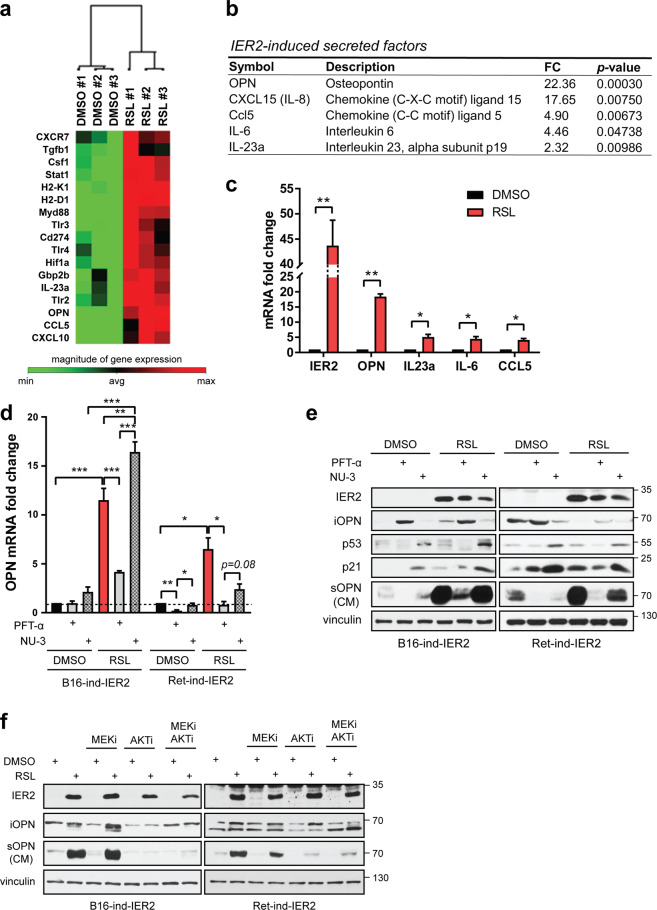
Osteopontin is a major component of the p53-dependent IER2-induced SASP. **a** Heatmap showing the top IER2-induced genes in B16-ind-IER2 #51 cells treated either with DMSO or RSL (3 × 0.5 μM) assessed using a Mouse Cancer Inflammation and Immunity Crosstalk PCR Array. **b** IER2-induced genes based on the qRT-PCR analysis (CXCL15, IL-6) and the Mouse Cancer Inflammation and Immunity Crosstalk PCR Array (OPN, Ccl5, and IL23a). Only genes with a fold change (FC) ≥ 2.0 and *p* < 0.05 are shown. See also Table S3. **c** qRT-PCR quantification of IER2, OPN, IL-23a, IL-6, and CCL5 in B16-ind-IER2 #51 cells treated with either DMSO or RSL (3 × 0.5 μM). RPLP0 was used for normalization. Bars represent means + SEM; *n* = 3. **d** qRT-PCR quantification of OPN in B16-ind-IER2 #51 and Ret-ind-IER2 #69 cells treated with PFT-α (20 μM) or NU-3 (5 μM) with or without stimulation with RSL (3 × 0.5 and 1 μM). RPLP0 was used for normalization in qRT-PCR. Bars represent mean + SEM; *n* = 3. **e** Western blot analysis of intracellular OPN (iOPN), IER2, p53, p21, vinculin (loading control), and secreted OPN (sOPN) present in conditioned media (CM) of B16-ind-IER2 #51 and Ret-ind-IER2 #69 cells treated with PFT-α (20 μM) or NU-3 (5 μM), with or without stimulation with RSL (3 × 0.5 and 1 μM). **f** Western blot analysis of intracellular OPN (iOPN), IER2, vinculin, and secreted OPN (sOPN) present in conditioned media (CM) of B16-ind-IER2 #51 and Ret-ind-IER2 #69 cells treated with or without RSL (3 × 0.5 and 0.5 μM) in the presence or absence of MEKi and/or AKTi (both 3 × 1 μM). Statistical significance was determined using an unpaired Student’s *t* test. *p < 0.05, **p < 0.01.

One of the hallmarks of senescence is the SASP [28, 53]. SASP factors can promote inflammation and support tumor cell invasion and metastasis [21, 54, 55]. We, therefore, reasoned that IER2 could conceivably promote melanoma progression through the induction of senescence and specific SASP components. To identify the components of the IER2-induced SASP in B16 cells, we initially employed a small-scale qRT-PCR screen of common SASP signaling factors. Although the expression of the core SASP cytokines IL-6 and CXCL15 (mouse homolog of *IL-8* [56]) was markedly upregulated, the expression of other SASP-associated factors tested including IL-1α, IL-1β, MMP3, CCL2, CXCL1, CXCL2, and CXCL5 was unaffected (data not shown). To identify other components of the IER2-induced SASP, we next employed a broader panel of 84 key genes involved in cancer inflammation and immunity (Fig. 4a, Table S3). The expression of 20 of these genes was significantly changed in response to IER2 expression, including genes previously identified in the transcriptomic analysis of IER2-expressing 3T3 cells (e.g., MYD88, TLR2, TLR3, TLR4, and IL-23a). Figure 4b summarizes genes that encode secreted proteins whose expression was increased more than twofold upon induction of IER2 expression. These data were validated by qRT-PCR (Fig. 4c), and also at the protein level for OPN (Fig. S4a). Together, these data suggest that the IER2-dependent SASP in B16 melanoma cells includes OPN, CXCL15, CCL5, IL-6, and IL-23a.

As OPN was by far the most highly upregulated gene in response to IER2 expression (Fig. 4a), and is known to play an important role in tumorigenesis and metastasis [57, 58], subsequent work focused on this gene. To explore the role of p53 in IER2-induced OPN expression, we treated IER2-inducible B16 and Ret cells with PFT-α and NU-3 and monitored OPN expression and secretion. Inhibition of p53 with PFT-α strongly suppressed the ability of IER2 to induce OPN transcription, while stabilization of p53 with NU-3 had no significant effect (Ret cells) or even enhanced OPN expression (B16 cells) (Fig. 4d). Similarly, the massive IER2-induced secretion of OPN (sOPN, secreted osteopontin) was strongly attenuated by inhibition of p53 with PFT-α, while it was maintained when cells were treated with NU-3 (Fig. 4e, for anti-OPN antibody specificity, see Fig. S4a).

To validate these findings in human melanoma cells, we ectopically expressed IER2 in p53^wt^ A375 cells, and in SK-Mel-28 cells, which carry a homozygous loss-of-function mutation in p53 (p53^L145R^) and therefore have only very low levels of p21 expression [59–61]. OPN expression and secretion, as well as the percentage of senescent cells, were significantly increased by ectopic expression of IER2 in p53^wt^ A375 cells, but not in p53^L145R^ SK-Mel-28 cells (Fig. S4b–d). In addition, the senescence phenotype in A375 cells was accompanied by activation of the AKT kinase and p53/p21 pathways, consistent with the results obtained in murine melanoma cells (Fig. 4e).

Since active ERK and AKT are important for IER2-induced p53 stabilization and senescence (Fig. 3b–e), we next examined whether inhibition of both pathways alone or in combination affects levels of the OPN protein. In contrast to the MEK inhibition, AKT inhibition resulted in a substantial decrease in secreted OPN upon IER2 expression both in B16 and Ret cells (Fig. 4f).

Together, these data indicate that sustained expression of IER2 can lead to a characteristic secretory phenotype in melanoma cells, a major component of which is OPN, which is upregulated in an AKT/p53-dependent manner.

### IER2-driven OPN is expressed by senescent cells

**Fig. 5:**
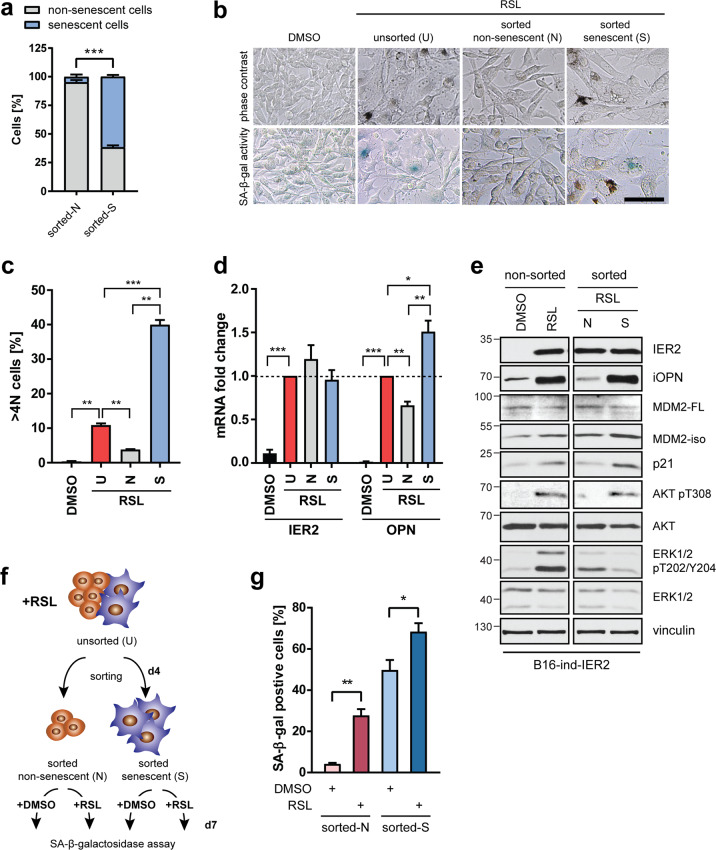
IER2 only induces osteopontin expression in senescent cells. **a** B16-ind-IER2 #51 cells were sorted into non-senescent (N) or senescent (S) cells, and enrichment of the corresponding types of cells in the sorted fractions was controlled by immediate post-sorting flow cytometry analysis. Bars represent means + SEM from three independent sorts. **b** Representative images showing cellular morphology and SA-β-galactosidase (SA-β-gal) activity (blue) in RSL-treated (3 × 0.5 μM) unsorted (U), sorted non-senescent (N) and sorted senescent (S) B16-ind-IER2 #51 cells. DMSO-treated cells were used as a control. Scale bar, 100 μm. **c** DRAQ5 labeling of nuclear DNA followed by flow cytometry quantification of >4 N B16-ind-IER2 #51 cells treated and sorted as in (**b**). See also Fig. S5c for the complete cell cycle profile. Bars represent mean + SEM, *n* = 3. **d**, **e** qRT-PCR quantification of IER2 and OPN (**d**) and Western blot analysis (**e**) of the indicated proteins in RSL-treated (3 × 0.5 μM) unsorted (U), sorted non-senescent (N) and sorted senescent (S) B16-ind-IER2 #51 cells. RPLP0 was used for normalization. DMSO-treated cells were used as a control. Bars in (**e**) represent mean + SEM; *n* = 5. **f** Schematic depiction of the sorting strategy for B16-ind-IER2#51 cells treated before and after sorting either with DMSO or RSL (3 × 0.5 μM). **g** B16-ind-IER2 #51 cells were treated for 3 days (3 × 0.5 μM) with RSL and sorted into non-senescent and senescent populations, each of which was subsequently treated for 3 days with DMSO or RSL (3 × 0.5 μM), followed by SA-β-gal staining. Bars represent mean + SEM; *n* = 4. Statistical significance was determined using an unpaired Student’s *t* test. *p < 0.05, **p < 0.01, ***p < 0.001.

The above results suggest that IER2 induces senescence stochastically, as senescence was only observed in around 10–25% of the cells (Figs. 2d, e, S4d). Therefore, we next assessed whether OPN is secreted by non-senescent (N) or senescent (S) cells after IER2 induction. We took advantage of the increased cellular size of senescent cells to sort them by flow cytometry-based on forward and side scatter (Fig. S5a, b). In line with previous results, IER2 induction markedly increased the percentage of enlarged senescent melanoma cells in the flow cytometry (Fig. S5b). The enrichment of senescent cells in the sorted fraction was confirmed by re-evaluation post-sorting (Fig. 5a), by monitoring of cellular morphology and SA-β-galactosidase activity (Fig. 5b), as well as by cell cycle analysis, including analysis of the percentage of >4 N cells (Figs. S5c, 5c). The senescence-enriched fraction also exhibited higher MDM2-iso and p21 levels compared to the non-senescent fraction (Fig. 5e), reflecting p53 activation. Although IER2 levels were elevated to the same extent in senescent and non-senescent cells, OPN was predominantly expressed by the senescent cell-enriched population (Fig. 5d, e). Notably, in contrast to the non-senescent fraction that showed higher levels of activated ERK1/2, the senescence-enriched fraction exhibited enhanced AKT activation (Fig. 5e). This is consistent with our observation that OPN secretion is abrogated by AKT inhibition but not ERK inhibition (Fig. 4f). Together, these data indicate that (i) the stochastic induction of senescence and OPN expression in response to IER2 is not due to variation in the expression level of IER2 within distinct subpopulations of cells, (ii) that enhanced IER2 expression does not induce senescence and OPN expression in all cells, and (iii) that OPN is part of the IER2-induced AKT/p53-dependent SASP.

To investigate whether the sorted non-senescent IER2-expressing B16 and Ret cells are resistant to IER2-induced senescence, they were exposed to an additional round of either DMSO or RSL treatment (Fig. 5f). IER2 induced senescence in these cells to the same extent (~25%) as in the non-sorted parental population (Fig. S5d, Fig. 5g). The sorted senescent cell-enriched fraction failed to revert to a non-senescent phenotype when IER2 expression was no longer maintained. Moreover, an additional round of RSL stimulation further increased the percentage of senescent cells in this fraction (Fig. S5d, Fig. 5g).

Overall, these data suggest that IER2 expression leads to stochastic senescence development in a fraction of melanoma cells, which elicits the production of OPN.

### IER2 nuclear localization is critical for senescence development and OPN secretion

**Fig. 6:**
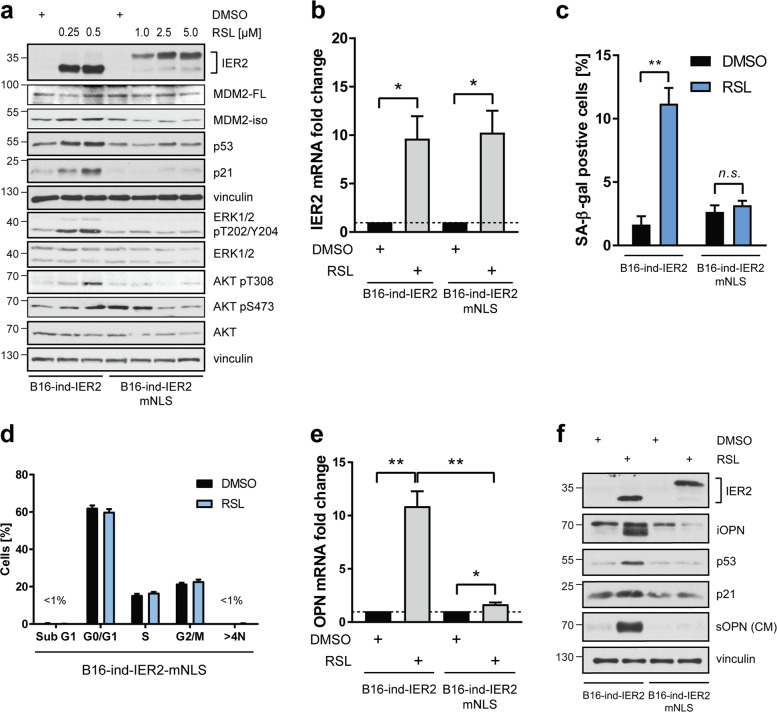
Nuclear localization of IER2 is required for senescence induction and osteopontin secretion. **a** Western blot analysis of the indicated proteins in B16-ind-IER2 #51 and B16-ind-IER2-mNLS #56 cells, treated with RSL at the indicated concentrations. DMSO was used as a control. **b** qRT-PCR quantification of IER2 in B16-ind-IER2 #51 (treated with DMSO or 0.25 μM RSL) and B16-ind-IER2-mNLS #56 cells (treated with DMSO or 5 μM RSL). RPLP0 was used for normalization. Bars represent mean + SEM; *n* = 4. **c** Quantification of SA-β-galactosidase (SA-β-gal)-positive B16-ind-IER2 #51 cells (treated with DMSO or 3 × 0.25 μM RSL) and B16-ind-IER2-mNLS #56 cells (treated with DMSO or 3 × 5 μM RSL). Bars represent mean + SEM; *n* = 3. **d** DRAQ5 labeling of nuclear DNA followed by flow cytometry-based cell cycle analysis of B16-ind-IER2-mNLS #56 cells after treatment with DMSO or RSL (3 × 5 μM). Bars represent mean + SEM; *n* = 3; 30,000 cells were analyzed per replicate. **e**, **f** qRT-PCR quantification of OPN (**e**) and Western blot analysis (**f**) of IER2, intracellular OPN (iOPN), p53, p21, vinculin, and secreted OPN (sOPN) in conditioned medium (CM) in B16-ind-IER2 #51 (treated with DMSO or 3 × 0.25 μM RSL) and B16-ind-IER2-mNLS #56 cells (treated with DMSO or 3 × 5 μM RSL). Bars represent mean + SEM; *n* = 4. Statistical significance was determined using an unpaired Student’s *t* test. n.s. nonsignificant (*p* > 0.05), *p < 0.05, **p < 0.01.

IER2 contains a putative bipartite NLS [9] that is conserved across species (Fig. S6a). To investigate whether the subcellular localization of IER2 is important for the induction of senescence and OPN secretion, we generated a RheoSwitch B16 cell line inducible for IER2 with a mutation in the first part of the putative NLS (IER2-mNLS, Fig. S6b). To be able to determine the effect of wild-type IER2 (IER2-wt) vs. mutated IER2-mNLS, we first titrated the RSL concentration required to achieve comparable induction of IER2 in both cell lines (Fig. 6a). Unexpectedly, IER2-mNLS exhibited altered electrophoretic mobility in sodium dodecyl sulfate-polyacrylamide gel electrophoresis (SDS-PAGE), producing two immunoreactive bands, indicative of possible changes in posttranslational modifications as a result of altered structure or cellular localization. Equivalent IER2 expression was further confirmed by qRT-PCR (Fig. 6b). Immunofluorescence staining of B16 cells expressing IER2-wt and IER2-mNLS confirmed that IER2-wt is partially localized in the nucleus, whereas IER2-mNLS is restricted to the cytoplasm (Fig. S6c).

In contrast to IER2-wt, IER2-mNLS induction failed to increase the levels of p53, MDM2-iso, and p21, or to activate the ERK and AKT kinases (Fig. 6a), and did not induce senescence (Fig. S6d, Fig. 6c) or result in alterations in the cell cycle (Fig. 6d). Furthermore, the nuclear localization of IER2 was crucial for the development of the IER2-induced SASP, as IER2-mNLS induced no, or only a much weaker upregulation of the IER2-wt-dependent SASP factors, including OPN (Figs. 6e, f, S6e).

Collectively, these data support the notion that the nuclear localization of IER2 is required for induction of senescence, as well as for production of the IER2-specific SASP that includes OPN.

### IER2 promotes melanoma migration and invasion via secreted OPN

**Fig. 7:**
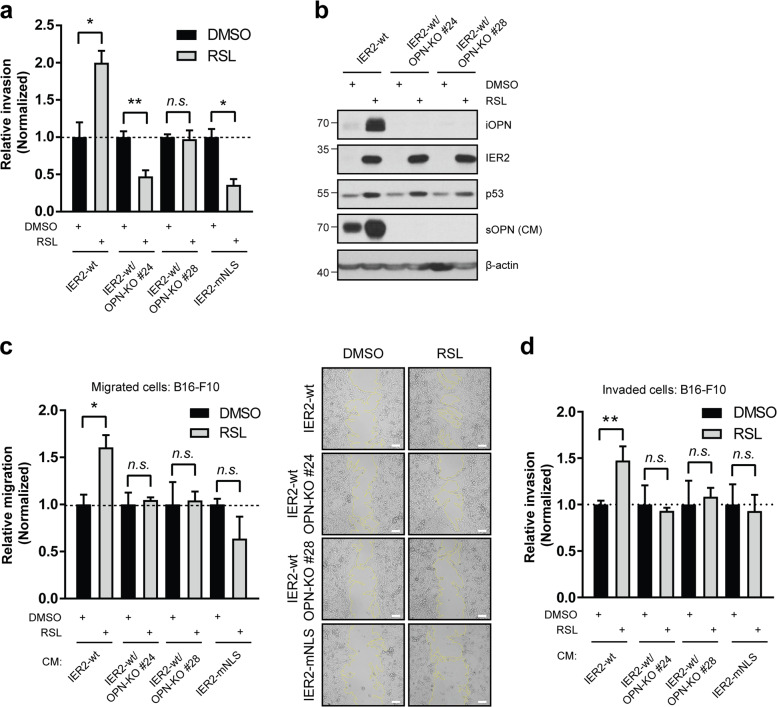
IER2 promotes melanoma cell migration and invasion in vitro in an osteopontin-dependent manner. **a** Invasion of B16-ind-IER2 #51, B16-ind-IER2-OPN-KO #24, B16-ind-IER2-OPN-KO #28, and B16-ind-IER2-mNLS #56 (IER2-mNLS) cells seeded in DMEM/1% FBS and treated either with DMSO or RSL (5 × 0.5 μM in the case of IER2-wt or 30 μM in the case of IER2-mNLS cells). Data represent relative invasion through a Matrigel layer (0.8 mg/ml) towards DMEM/10% FBS at 64 h and are shown as mean + SEM; *n* = 3. Data are normalized to DMSO-treated controls. **b** Western blot analysis of intracellular OPN (iOPN), IER2, p53, β-actin (loading control), and secreted OPN (sOPN) in conditioned medium (CM) in B16-ind-IER2 #51 (IER2-wt) cells, as well as in B16-ind-IER2-OPN-KO #24 (IER2-wt/OPN-KO #24) and B16-ind-IER2-OPN-KO #28 (IER2-wt/OPN-KO #28) cells, in which osteopontin was deleted through CRISPR/Cas9-mediated gene inactivation, treated either with DMSO or RSL (3 × 0.5 μM). **c** Migration of wild-type B16–F10 cells 32 h after removal of silicone inserts in the presence of CM from B16-ind-IER2 #51, B16-ind-IER2-OPN-KO #24, B16-ind-IER2-OPN-KO #28, and B16-ind-IER2-mNLS #56 cells treated either with DMSO or RSL (3 × 0.5 μM in case of IER2-wt or 30 μM in case of IER2-mNLS cells). Data represent relative migration + SEM from 3 to 4 biological replicates normalized to DMSO-treated controls. Scale bar, 100 μm. **d** Invasion of wild-type B16–F10 cells in the presence of CM from B16-ind-IER2 #51, B16-ind-IER2-OPN-KO #24, B16-ind-IER2-OPN-KO #28, and B16-ind-IER2-mNLS #56 cells that were seeded in DMEM/1% FBS and treated either with DMSO or RSL (5 × 0.5 μM in the case of IER2-wt or 30 μM in the case of IER2-mNLS cells). Data represent relative invasion through a Matrigel layer (0.8 mg/ml) toward DMEM/10% FBS at 48 h and are shown as mean + SEM; *n* = 3. Data are normalized to DMSO-treated controls. n.s. nonsignificant (*p* > 0.05), *p < 0.05, **p < 0.01, with significance determined by an unpaired Student’s *t* test.

We have previously reported that IER2 is rapidly translocated to the nucleus of actively migrating pancreatic tumor cells after relief of contact inhibition [13]. Therefore, we next assessed whether the nuclear localization of IER2 impacts the migration of B16 cells. The proliferation of B16 cells expressing IER2-wt was lower than that of IER2-mNLS-expressing cells (Fig. S7a). However, the migration of cells expressing IER2-wt was enhanced in comparison to IER2-mNLS-expressing cells (Fig. S7b). We also investigated whether the nuclear localization of IER2 promotes melanoma invasion. Again, expression of IER2-wt strongly promoted melanoma cell invasion, whereas expression of IER2-mNLS even suppressed invasion, rather than promoting it (Fig. 7a).

To dissect the role of OPN in IER2-mediated invasion, we generated IER2-inducible B16 cells with CRISPR/Cas9-mediated knockout of OPN (OPN-KO, Fig. 7b, Fig. S7f). These cells retained the ability to induce p53 and to develop a senescence phenotype upon IER2 induction (Figs. 7b, S7c, d). IER2 expression, as well as the absence of OPN, had no effect on proliferation in these cells (Fig. S7e). By contrast, deletion of OPN completely abrogated the ability of wild-type IER2 to promote melanoma cell invasion (Fig. 7a, S7f, g).

Senescent fibroblasts can stimulate the invasiveness of malignant epithelial cells via paracrine signaling [62]. Therefore, we next assessed whether conditioned medium (CM) from IER2-expressing B16 cells can affect the migration and invasion of parental, non-senescent B16 cells. CM from IER2-wt-expressing cells robustly induced the migration and invasion of parental B16 cells (Fig. 7c, d). By contrast, CM from IER2-mNLS-expressing cells or from IER2-wt-expressing cells with a knockout of OPN (OPN-KOs) had no impact on the migration and invasion of parental, non-senescent B16 cells (Fig. 7c, d). Interestingly, CM from IER2-wt-expressing B16 cells had no effect on the migration, invasion or soft agar growth of normal melanocytes (Fig. S8a–d), demonstrating that the IER2-driven secretome is not sufficient to modify the transformation status or invasive behavior of non-malignant melanocytes.

Together, these results demonstrate that IER2 fosters melanoma cell migration and invasion in a manner that requires nuclear localization of the IER2 protein. IER2-induced OPN expression mediates these effects and can act in a paracrine manner on neighboring, non-senescent cancer cells.

### IER2 levels correlate with OPN expression in human melanoma

**Fig. 8:**
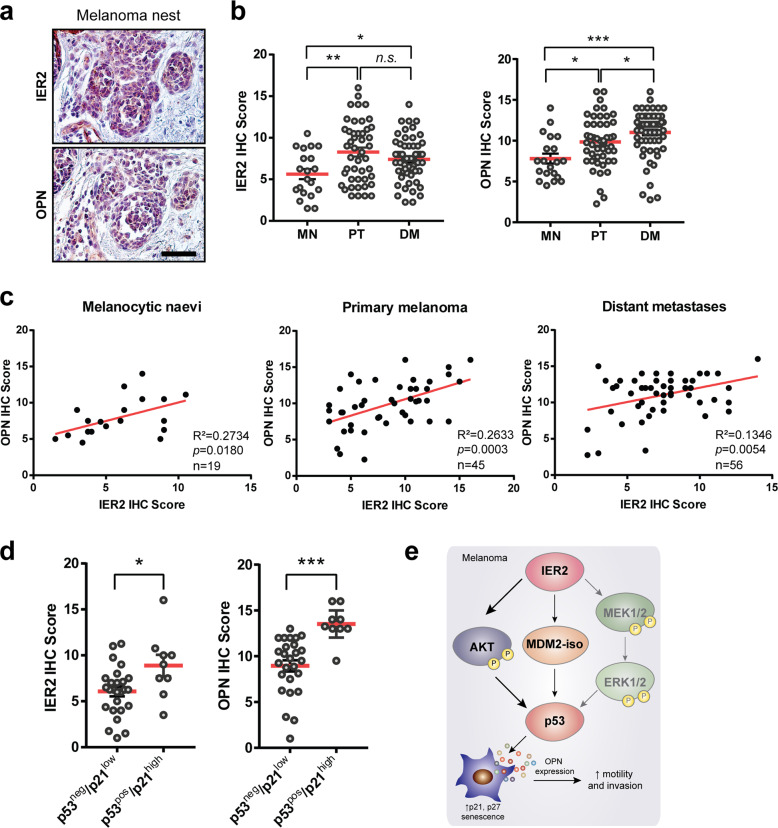
High IER2 levels are associated with increased osteopontin levels and p53/p21 expression in human melanoma samples. **a** Representative images of serial sections of melanoma nests (Clark level IV) stained with IER2 and OPN. Scale bar, 50 μm. **b** TMA immunohistochemistry (IHC) scores for IER2 (left) and OPN (right) for melanocytic nevi (MN, *n* = 20), primary tumors (PT, *n* = 46 for IER2 and 48 for OPN), and distant metastases (DM, *n* = 56 for IER2 and 63 for OPN). Data represent mean + SEM. **c** Correlation analysis of IER2 and OPN expression-based IHC analysis of human melanoma TMAs stratified into melanocytic nevi, primary melanomas, and distant metastases. The coefficient of determination (*R*^2^) of linear regression analysis, the *p* value of Pearson correlation analysis, and the number of samples analyzed are indicated for each graph. **d** TMA immunohistochemistry (IHC) scores for IER2 (left) and OPN (right) in specimens stratified according to p53/p21 status (inactive: p53^neg^/p21^low^, *n* = 26; active: p53^pos^/p21^high^, *n* = 9). **e** Scheme illustrating molecular functions of IER2 in melanoma. Data represent mean + SEM. n.s. nonsignificant (*p* > 0.05), *p < 0.05, **p < 0.01, ***p < 0.001. In **b** and **d**, statistical significance was determined using an unpaired Student’s *t* test.

To gain insight into the association between the IER2-p53 axis and OPN expression in human melanoma, we first performed immunohistochemistry analysis of IER2 and OPN expression in tissue biopsies from patients with primary melanomas. Immuno-staining of serial sections revealed co-expression of IER2 and OPN in melanoma nests (Fig. 8a). To investigate the relationship between IER2 and OPN expression during melanoma progression, we analyzed tissue microarrays (TMAs) containing human melanocytic nevi (MN), primary melanomas (primary tumors, PT), and distant melanoma metastasis (DM). Expression intensity and abundance were assessed in a blinded fashion by two independent researchers using a defined immunohistochemical score for each probe (Fig. S9a). This analysis demonstrated a significant upregulation of both IER2 and OPN protein expression in primary melanomas, compared to MN, and further upregulation of OPN in distant metastases (Fig. 8b, for clinicopathological data, see Table S4). These observations are consistent with previous reports showing that OPN expression correlates with melanoma invasion [63]. Furthermore, our TMA analysis revealed a significant correlation between IER2 and OPN protein expression in MN, PT, and distant metastases (Fig. 8c, for clinicopathological data, see Table S4).

We next aimed to determine whether IER2 and OPN expression is linked to a p53/p21-driven senescence-like state in melanomas, as suggested by our results using melanoma cell lines. Since the paraffin embedding used for the TMA samples destroys β-galactosidase activity, we used p53 status combined with p21 expression (p53^pos^/p21^high^ versus p53^neg^/p21^low^, see Fig. S9b, c for IHC staining) as a surrogate marker of senescent cells. Importantly, both IER2 and OPN showed significantly higher expression in patients with an active p53–p21 pathway (p53^pos^/p21^high^), consistent with our findings that demonstrate a functional link between IER2, p53, and OPN (Fig. 8d, for clinicopathological information, see Table S5).

Taken together, these data provide evidence that the p53-dependent IER2-induced senescence and OPN expression, we observed in cultured melanoma cells, reflects the situation in human melanoma.

## Discussion

Here, we report that high levels of IER2 in human melanoma samples correlate with increased p53/p21 and OPN levels, as well as with poor patient prognosis. Mechanistically, we show that IER2 upregulates p53 protein expression via increased expression of MDM2-iso and enhanced activation of AKT and ERK1/2, which leads to the stochastic induction of senescence in melanoma cells. The senescent cells express a characteristic SASP, a major component of which is OPN. OPN from the senescent cells acts in a paracrine manner to promote motility and invasion of non-senescent melanoma cells (Fig. 8e).

Our data suggest that constitutive expression of IER2 on the one hand leads to accumulation of MDM2-iso, and on the other to increased activation of AKT and ERK1/2, all of which leads to stabilization of p53 and expression of p21 (Fig. 8e). These data are consistent with the induction of premature senescence that is observed in response to constitutive MEK/MAPK signaling [64], and with the DNA damage-independent increase in p53 levels and senescence that are induced via mTORC1 in response to PI3K/AKT pathway activation [51]. Activation of AKT, which can be promoted by IER2 [17], also increases intracellular ROS levels by inducing oxygen consumption or by inhibiting the forkhead box O (FOXO) family of transcription factors, which further fosters cellular senescence [65, 66].

Our data indicate that OPN is a major component of the IER2-induced SASP in melanoma. OPN has previously been identified in some but not all SASPs in a context-dependent manner. OPN is a component of the bleomycin-induced senescence SASP that is produced independently of DNA damage, and can also be produced by senescent stromal cells within the tumor microenvironment [67, 68]. OPN is additionally produced by senescent pulmonary artery smooth muscle cells and contributes to the progression of pulmonary hypertension [69]. Context-dependent inclusion of OPN in the SASP presumably reflects the fact that multiple signaling pathways regulate the SASP, including the DDR, p38 MAP kinase, and cGAS/STING pathways [70]. Our data indicate that AKT plays an important role in regulating the IER2-induced SASP that includes OPN, which is consistent with the observation that the downstream AKT target mTOR controls the senescent secretome through regulating the stability of SASP mRNAs [71]. In bleomycin-induced senescence, the transcription factors c-Myb and C/EBPb drive OPN expression [72]. OPN expression in bleomycin-treated senescent fibroblasts is independent of p53 and Rb [68], but our results show that the IER2-induced SASP, including OPN expression, is p53 dependent in melanoma cells, which is consistent with the direct activation of OPN transcription by p53 [73].

The association between IER2, p53/p21, and poor prognosis in melanoma patients suggests that IER2-induced senescence contributes to tumor progression. This could be mediated at a number of levels. There is increasing evidence that subpopulations of senescent tumor cells may be able to revert to a proliferative state [74]. Senescent tumor cells that resume proliferation have been reported to be more aggressive [26], and to be reprogramed into self-renewing, tumor-initiating cells [75]. Furthermore, the IER2-induced SASP in the senescent subpopulation of cells could conceivably stimulate the growth and aggressiveness of non-senescent tumor cells. In particular, OPN secreted into the tumor microenvironment has been reported to promote tumor growth and metastasis in a number of ways. Through activating receptors such as integrins and CD44, OPN can foster survival, proliferation, motility, and invasion of tumor cells, and can stimulate angiogenesis [58]. Consistently, we found that OPN in the IER2-induced SASP produced by senescent melanoma cells is able to stimulate the motility and invasion of non-senescent melanoma cells in a paracrine manner. This is in line with a large body of prior work demonstrating a pro-invasive effect of OPN on tumor cells, mediated by a diverse set of molecular mechanisms [58]. Furthermore, OPN can also recruit myeloid-derived suppressor cells and other bone marrow-derived cells to the tumor microenvironment and metastatic niches, thereby suppressing anticancer immunity and promoting metastasis [76–78]. Moreover, OPN can reprogram normal fibroblasts into tumor-promoting cancer-associated fibroblasts [79].

IER2 could conceivably induce senescence through several mechanisms. Our data clearly show that IER2-induced senescence and OPN production is p53-dependent and requires nuclear localization of the IER2 protein. This is consistent with a proposed transcriptional regulatory role of IER2 [11], with putative IER2 target genes possibly activating senescence-inducing pathways. IER2 can also regulate the substrate specificity of phosphatase PP2A [12]. As PP2A activity has been implicated in the induction of senescence [80, 81], this suggests a possible further mechanism through which IER2 might induce senescence. In addition, our time-lapse data suggest that IER2-induced senescence may be associated with incomplete or delayed cytokinesis (Video S1). Defective cytokinesis can increase p53 levels via proteolytic cleavage of MDM2 [82], induce senescence [83, 84], and result in tetraploidy [85], all of which are features we observed upon continuous induction of IER2 expression. It is, therefore, conceivable that constitutive IER2 expression may perturb cytokinesis and thereby induce senescence. In this regard, we note that the AKT pathway, which we show is activated by IER2, is involved in context-dependent cytokinesis failure [86]. Defective cytokinesis can also result in aneuploidy in cancer cells, which fosters genomic instability that can drive tumorigenesis [85, 87] and thus may represent a further mechanism through which IER2-induced senescence might conceivably promote cancer progression if cells are subsequently able to re-enter the cell cycle.

IER2 induces senescence in only a subpopulation of melanoma cells, despite the fact that all cells induced with RSL express IER2 (Fig. 5e). This could conceivably reflect heterogeneity in the melanoma cell populations, yet this seems unlikely, as the IER2-inducible cells underwent two rounds of single-cell cloning before experiments were performed. The production of SASP components above a given threshold by individual cells may be decisive, as several SASP components can establish and reinforce the senescence phenotype through autocrine positive feedback loops [88–90]. In addition, recently published single-cell tracking experiments suggest that heterogeneity in the decision of whether or not cells enter senescence is determined by differences in the dynamics of p21 expression at particular cell cycle phases [91]. Similarly, the stochastic induction of senescence has been associated with the kinetics of p53 expression, with pulsed p53 expression resulting in reversible arrest, while continuous p53 expression results in senescence [92]. Thus the stochastic induction of senescence upon continuous IER2 expression that we observed may be a consequence of cell fate decisions that are made in response to the kinetics of expression of key regulatory proteins such as p21 and p53 at particular phases of the cell cycle. This notion would be consistent with the observation that a proportion of sorted non-senescent cells that have expressed IER2 constitutively over at least two cell cycles can nevertheless still be induced to enter senescence upon continued IER2 induction (Fig. 5f, g).

Our findings may have clinical relevance at a number of levels. We found that increased IER2 expression is associated with high p53/p21 levels in human melanoma samples, which would be consistent with the induction of senescence observed in vitro. Furthermore, IER2 expression is associated with enhanced OPN expression in human melanoma and also correlates with poor prognosis. IER2, the IER2-induced SASP including OPN, as well as IER2-induced senescent cells themselves may therefore represent therapeutic targets in melanoma. Given the extensive literature documenting the tumor-promoting role of OPN, blocking the activity of the OPN protein or inhibiting activation of its receptors using small molecules have been suggested as possible therapeutic approaches [93]. As OPN has been implicated in fostering chemoresistance, possibly through autophagy or suppressing apoptosis [58], IER2-induced OPN expression may also represent a mechanism that fosters resistance to chemotherapy. Furthermore, senescent cells exhibit resistance to apoptosis [70], and IER2-induced senescence may therefore render melanoma cells intrinsically resistant to chemotherapy-induced apoptosis, as well as through the production of OPN in the SASP. This may be relevant to the development of therapy resistance, as transient chemotherapy-induced senescence has been reported to render cancer cells more aggressive if they are able to subsequently re-enter the cell cycle [26, 27]. Together, these observations suggest that targeting IER2-induced senescent melanoma cells may improve therapeutic outcomes. A number of senolytic drugs that aim to clear senescent cells by inducing apoptosis are currently under development, and the first is already in clinical trials [94].

In summary, these data suggest that aberrant activation of the IER2–p53–OPN axis in melanoma represents a pathophysiological mechanism that can contribute to the aggressive behavior of melanoma cells.

## Materials and methods

### Cell lines and molecular cloning

The human melanoma cell lines A375, SK-Mel-28, and HT144 as well as NIH 3T3 mouse fibroblasts (3T3) and B16-F10 mouse melanoma cells (B16) were all from American Type Culture Collection (ATCC, Manassas, VA, USA). SK-Mel-147 and SK-Mel-23 were from Memorial Sloan Kettering Cancer Center (NY, USA). Mouse melanoma Ret cells [95] were provided by V. Umansky. 67NR mouse mammary carcinoma cells [96] were established by Dr. Fred Miller and kindly provided by E. Lukanidin (Danish Cancer Society, Copenhagen, Denmark). NIH 3T3 RheoSwitch cells (clones 3T3-ind-IER2 #11, #19, and #22) have been described previously by us [31]. All melanoma and fibroblast cell lines were cultivated in DMEM containing 4.5 g/l glucose (Gibco, Carlsbad, CA, USA) supplemented with 10% fetal bovine serum (FBS; Sigma Aldrich, St. Louis, MO, USA) and 1% penicillin/streptomycin sulfate (Gibco, Carlsbad, CA, USA). Mel-STV human melanocytes [97] were cultured in DMEM supplemented with 5% FBS and 1% penicillin/streptomycin. Melan-a murine melanocytes (Merck, Darmstadt, Germany) were cultured in RPMI 1640 medium (Gibco, Carlsbad, CA, USA) supplemented with 10% FBS and 200 nM phorbol-12-myristate-13-acetate (AdipoGen, San Diego, CA, USA). Cells were kept at 37 °C in an atmosphere with 5% CO_2_ and 95% humidity. The human cancer cell lines were authenticated by the vendor. Mycoplasma contamination was routinely checked in the laboratory by PCR analysis and all cell lines used in the study were confirmed to be mycoplasma negative.

The RheoSwitch two-plasmid system for inducible expression of mammalian genes was purchased from New England Biolabs (Ipswich, MA, USA). pcDNA3.1-empty and pcDNA3.1-TP53 were provided by Dr. Takashi Tokino [73]. The pcDNA3.1/V5-His-IER2 plasmid was generated previously [13] by cloning human IER2 cDNA into pcDNA3.1/V5-His empty vector (Invitrogen, Thermo Fischer Scientific, Waltham, MA, USA). All constructs were verified by sequencing.

B16-ind-IER2 #51, Ret-ind-IER2 #69, 67NR-ind-IER2 #37, and 67NR-ind-IER2 #82 cell clones were prepared by transfection of RheoSwitch B16-pNEBR-R1, Ret-pNEBR-R1, and 67NR-pNEBR-R1 cells with a pNEBR-X1-IER2 plasmid, containing cloned mouse IER2 cDNA [31]. B16-ind-IER2-mNLS #56 cells were prepared by transfection of RheoSwitch B16-pNEBR-R1 cells with a pNEBR-IER2 plasmid, containing IER2 cDNA mutated in the NLS using the Stratagene QuikChange Site-Directed Mutagenesis Kit (Agilent Technologies, Santa Clara, CA, USA) according to the manufacturer’s instructions. All cells were transfected using Lipofectamine2000 (Thermo Fisher Scientific, Waltham, MA, USA) following the manufacturer’s instructions, and selected with G418 (500–700 µg/ml; Roche Applied Science, Penzberg, Germany) and hygromycin (500 µg/ml; Merck, Darmstadt, Germany) as appropriate. Single-cell colonies were expanded and tested for IER2 expression.

For live cell imaging, 3T3-IER2-H2B-GFP cells were generated as follows. IER2-inducible 3T3 #11 cells [98] were stably transfected with the pBOS-H2B-GFP expression plasmid (BD Pharmingen, New Jersey, USA). Live cell imaging was performed using an Olympus IX50 inverted microscope equipped with an F-View II imaging system and Cell^P imaging software (Olympus, Shinjuku, Tokyo, Japan).

B16-ind-IER2-OPN-KO #24, #28, #33, and #36 cells were generated by CRISPR/Cas9-mediated targeting of OPN, following published protocols [99]. Briefly, One-Shot Stbl3 Chemically Competent *Escherichia coli* cells (Invitrogen, Thermo Fischer Scientific, Waltham, MA, USA) were transformed with pSpCas9(BB)-2A-Puro V2.0 plasmid (Addgene, #62988), containing three sgRNA pairs, designed at http://crispr.mit.edu/, targeting the mouse osteopontin (Spp1) locus. B16-ind-IER2 #51 cells were transfected with sequence-verified plasmids and pulse-selected with puromycin for 72 h (InvivoGen, San Diego, CA, USA). Cells were tested for osteopontin expression using qRT-PCR and Western blotting. The top-scoring sgRNA pair (sgRNA-top: 5′-cac cgA AGC TAT CAC CTC GGC CGT T-3′; sgRNA-bottom: 5′-aaa cAA CGGC CGAG GTGA TAGC TTc-3′) was used to generate single-cell clones.

### Chemicals and cell treatments

Cells were treated with the following reagents at the indicated concentrations. Doxorubicin hydrochloride (Doxo, 2 μM) and camptothecin (CPT, 2 μM) were purchased from Sigma-Aldrich/Merck (Darmstadt, Germany). Nutlin-3(a) (NU-3, 5 μM) and Pifithrin-α (PFT-α, 30 μM) were purchased from Biomol (Hamburg, Germany). RheoSwitch Ligand 1 (RSL, 0.2–30 μM) was purchased from New England Biolabs (Ipswich, MA, USA) and Exclusive Chemistry Ltd. (Obninsk, Russia). The MEK/ERK inhibitor Selumetinib (AZD6244; MEKi, 1 μM) was purchased from Selleckchem (Münich, Germany) and the AKT inhibitor (AKTi, 1 μM) from Sigma-Aldrich/Merck, #A6730 (Darmstadt, Germany). 3T3 cells were irradiated with 30 J/m^2^ UV-C (245 nm) and cells were harvested 1 h after the irradiation.

### SDS-PAGE and Western blotting

Cells were washed twice with phosphate-buffered saline (PBS), harvested into hot SDS sample lysis buffer (4% SDS, 125 mM Tris-HCl pH 6.8, 20% glycerol in double-distilled H_2_O) containing Complete Protease Inhibitor Cocktail (Roche Applied Science, Penzberg, Germany), and sonicated using a Soniprep 150 disintegrator (MSE, London, UK). Protein concentration was determined using the BCA Protein Assay Kit (Pierce Biotechnology, IL, Rockford, USA). DTT (100 mM final concentration) and 0.01% bromphenol blue were added to lysates before separation by SDS-PAGE. The same protein amount (30–50 μg) was loaded into each well. Proteins were electrotransferred onto a nitrocellulose membrane (GE Healthcare, Little Chalfont, UK) at 4 °C overnight using wet transfer. Specific proteins were detected using primary antibodies (overnight incubation at 4 °C) followed by horseradish peroxidase-conjugated secondary antibodies (2–4 h incubation at 4 °C). Peroxidase activity was detected by enhanced chemiluminescence (ECL; Pierce Biotechnology, Rockford, IL, USA). GAPDH, vinculin, or β-actin were used as loading controls.

To detect secreted osteopontin (sOPN), proteins in 5 ml conditioned media were incubated with 10 μl StrataClean Resin (Agilent Technologies, Santa Clara, CA, USA) and rotated for 1–2 h at 4 °C. After brief centrifugation, the pellet was resuspended in lysis buffer (125 mM Tris-HCl pH 6.8, 0.2% SDS, 20% glycerin, and 1× Complete Protease Inhibitor Cocktail) and samples were incubated for 5 min at 95 °C. Lysates were adjusted to 100 mM DTT and 0.01% bromphenol blue, then 10 μg of total protein (measured with the BCA method) were loaded onto SDS-PAGE gels and Western blotted.

The following antibodies were used for immunoblotting: anti-mouse **IER2** (clone 1A2, [13]); anti-human **IER2** (ARP34401_P050) from Aviva Systems Biology (San Diego, CA, USA); **osteopontin** (OPN, #AF808) from R&D Systems (Mineapolis, MN, USA); **vinculin** (#V9131), **MDM2** (3G9, 04–1530), and **β-actin** (AC-15, #A5441) from Merck (Darmstadt, Germany); **p16**^**INK4A**^ (p16, ab189034) and **p21**^**WAF1/Cip1**^ (p21, EPR3993, ab109199) from Abcam (Cambridge, MA, USA); **p27**^**KIP1**^ (p27, F-8, sc-1641), **p21**^**WAF1/Cip1**^ (p21, C-19, sc-397), **ERK1** (ERK, C-16, sc-93), **AKT1/2/3** (AKT, H136, sc-8312), **MDM2** (SMP14, #sc-965), and **p53** (DO-7, sc-47698) from Santa Cruz Biotechnology (Dallas, TX, USA); **p53** (1C12, #2524), **p53-pS15** (D4S1H, #12571), **Rb-pS807/811** (D20B12 XP), **Rb** (D20), **AKT-pS473** (587F11, #4051), **AKT-pT308** (D25E6, #13038), **ERK1/2-pT202/pY204** (#9101), **GAPDH** (14C10, #2118), **mTOR** (#2972) and **mTOR-pS2448** (#2971) from Cell Signaling Technology (Danvers, MA, USA); and **p53-pS392** (FP3.2, #629501) from BioLegend (San Diego, CA, USA). Anti-rabbit and anti-mouse IgG-HRP secondary antibodies produced in goat, and anti-goat secondary antibody produced in rabbit were purchased from Dako (Hamburg, Germany). Antibodies were diluted in PBS containing 2.5% skimmed milk and 0.1% Tween (Carl Roth, Karlsruhe, Germany) according to the respective manufacturer’s instructions. Quantification of Western blots was performed in ImageJ/Fiji [100]. Protein expression was calculated as the integrated density of each band normalized to the average of control-treated samples.

### Quantitative real-time PCR (qRT-PCR)

Total RNA was isolated using TRIzol reagent (Thermo Fisher Scientific, Waltham, MA, USA), following the manufacturer’s instructions. RNA (1–2 μg) was treated with RNase-free DNase I, followed by EDTA deactivation for 10 min at 65 °C (both from Thermo Fisher Scientific, Waltham, MA, USA). First-strand cDNA was synthesized with random hexamer primers, using dNTP mix and RevertAid H Minus Reverse transcriptase (all from Thermo Fisher Scientific, Waltham, MA, USA). qRT-PCR was performed in a Stratagene Mx3500P qPCR machine (Agilent, Santa Clara, CA, USA) using SYBR Select Master Mix containing SYBR Green dye (Applied Biosystems, Foster City, CA, USA). The relative quantity of cDNA was estimated using the ΔΔCT method, and data were normalized to RPLP0 or GAPDH.

The following mouse forward and reverse primers, purchased from Metabion (Steinkirchen, Germany), were used for qRT-PCR: **IER2:** 5′-TAC CTC TCA GCC AAG GTA GA-3′, 5′-TCC TCT TGC GTA TCC ATG GG-3′; **IL-6**: 5′-TAC CAC TTC ACA AGT CGG AGG C-3′, 5′-CTG CAA GTG CAT CAT CGT TGT TC-3′; **CXCL15:** 5′-GGT GAT ATT CGA GAC CAT TTA CTG-3′, 5′-GCC AAC AGT AGC CTT CAC CCA T-3′; **OPN:** 5′-GCT TGG CTT ATG GAC TGA GGT C-3′, 5′-CCT TAG ACT CAC CGC TCT TCA TG-3′; **IL23a:** 5′-CAT GCT AGC CTG GAA CGC ACA T-3′, 5′-ACT GGC TGT TGT CCT TGA GTC C-3′; **CCL5:** 5′-CCT GCT GCT TTG CCT ACC TCT C-3′, 5′-ACA CAC TTG GCG GTT CCT TCG A-3′; **TP53**: 5′-GCA TGA ACC GCC GAC CTA TCC-3′, 5′-CAG GGC AGG CAC AAA CAC GAA C-3′; **RPLP0**: 5′-GGA CCC GAG AAG ACC TCC TT-3′, 5′-GCA CAT CAC TCA GAA TTT CAA TGG-3′.

The following human forward and reverse primers, purchased from Metabion (Steinkirchen, Germany) were used for qRT-PCR: **IER2**: 5′-AGT GCA GAA AGA GGC ACA GC-3′, 5′-ACC TTG GCC GAG AGG TAG AG-3′; **OPN**: 5′-CTG ACA TCC AGT ACC CTG ATG C-3′, 5′-GGC CTT GTA TGC ACC ATT CA-3′; **TP53**: 5′-CAG CAC ATG ACG GAG GTT GT-3′, 5′-TCA TCC AAA TAC TCC ACA CGC-3′; **GAPDH:** 5′-CGA CCA CTT TGT CAA GCT CA-3′, 5′-AGG GGT CTA CAT GGC AAC TG-3′.

The data are expressed as the means + SEM from 3 to 5 biological replicates, analyzed in technical duplicates.

### RNA interference

Small interfering RNAs (siRNA) targeting mouse OPN (siOPN, s74322, #4390771) were purchased from Thermo Fisher Scientific (Waltham, MA, USA). Silencer Select Negative Control No. 1 siRNA (Thermo Fisher Scientific, #4390843) was used as a control. Mouse Trp53 was targeted using ON-TARGETplus SMARTpool siRNA (L-040642-00-0005) and ON-TARGETplus Non-targeting Control Pool siRNA (D-001810-10-05) as control (Horizon Discovery, Cambridge, UK). OPN siRNAs were introduced into the cells using Lipofectamine RNAiMAX (Invitrogen, Carlsbad, CA, USA), while Trp53 siRNAs were transfected at a final concentration of 25 nM using Lipofectamine 2000 (Thermo Fisher Scientific, Waltham, MA, USA), following the manufacturer’s instruction.

### Cytokine array

Gene expression was analyzed in three independent experiments using the mouse Cancer Inflammation and Immunity Crosstalk Array (PAMM-181Z, Qiagen Sciences, Germantown, MD, USA) according to the manufacturer’s instructions.

### Indirect immunofluorescence

Cells grown on glass coverslips were washed with PBS, fixed with 100% ice-cold methanol (AppliChem, Darmstadt, Germany) for 10 min at −20 °C, then permeabilized using 0.1% Triton X-100 (Carl Roth, Karlsruhe, Germany) for 10 min at room temperature (RT). After washing with PBS, cells were incubated in 10% FBS/PBS for 30 min to block unspecific binding. Cells were incubated with IER2 antibody (ARP34401_P050; Aviva Systems Biology, San Diego, CA, USA) diluted 1:500 in blocking solution for 3 h at RT, then extensively washed with PBS. Incubation with the goat anti-rabbit AlexaFluor 546 (Thermo Fisher Scientific, Waltham, MA, USA) secondary antibody (1:1000) was performed for 1 h at RT. Cell nuclei were counterstained with 1 μg/ml DAPI (AppliChem, Darmstadt, Germany) for 5 min at RT, and coverslips were mounted in Fluoromount mounting medium (SouthernBiotech, Birmingham, AL, USA). Fluorescent signals were captured using a Zeiss Axio Imager D1 microscope equipped with an AxioCam MRm camera and AxioVision 4.7 software (Carl Zeiss, Oberkochen, Germany).

### Immunohistochemistry

Formalin-fixed, paraffin-embedded 5 μm-thick tumor sections were used for immunohistochemical staining. Deparaffinization in Roti-Histol (Carl Roth, Karlsruhe, Germany) and dehydration in 100–70% EtOH was followed by 30 min heat-induced antigen retrieval (Target Retrieval Solution pH 6.1; Dako, Hamburg, Germany). Endogenous peroxidase was quenched with hydrogen peroxide (3%, 5 min, Roth, Karlsruhe, Germany), and unspecific antibody binding was blocked using 10 % goat or rabbit serum in 1% BSA/PBS for 1 h at RT. Samples were then incubated with **IER2** (ARP-34401_P050; 1:500) from Aviva Systems Biology (San Diego, CA, USA); **p53** (DO-7; 1:150) and **p21**^**WAF1/Cip1**^ (p21, C19; 1:800) from Santa Cruz Biotechnology (Dallas, TX, USA), and **OPN** antibody (AF808; 1:100) from R&D Systems (Minneapolis, MN, USA) at 4 °C overnight. After washing 3x with PBS, slides were incubated with biotinylated secondary antibody for 1 h at RT (Dako, Hamburg, Germany). Antibody staining was detected using the VECTASTAIN ABC kit and the VECTOR NovaRed peroxidase substrate kit (both from Vector Laboratories, Burlingame, CA, USA). Sections were counterstained with hematoxylin (Merck, Darmstadt, Germany). All primary antibodies were titrated and specificity-tested through negative controls (without primary antibody) prior to their use for TMAs. Tumors derived from MMTV-PyMT mice were used as a positive control for the anti-osteopontin antibody. Visualization of melanoma cells was performed by staining against S100B (Abcam, Cambridge, UK) as described [101].

For scoring of IER2-, OPN-, and p21-stained TMAs, a quantity-/intensity-based IHC scoring system was applied, using the following formula: percentage of IER2/OPN positive cells among the S100B-positive cells (0–4) × intensity score (0–4) (see Fig. S8A). Nuclear positivity for p53 was assessed based on the percentage of p53-positive cells per punch using a 10% cutoff (≤10% tumor cells staining = negative, ≥10% = positive) as previously described [102]. Images of stained tumor sections and TMAs sections were captured using a Zeiss Axio Imager Z1 microscope equipped with an AxioCam HRc camera and AxioVision 4.8 software (Carl Zeiss, Oberkochen, Germany). Image acquisition and scoring were performed in a blind fashion by two independent experimenters (L.K. and S.D.S.), in the majority of cases from duplicate stainings. Low-quality IER2- and OPN-stained specimens were excluded from the analysis, which resulted in differing total numbers of specimens analyzed. For each sample, the average score of the two experimenters was used.

### Flow cytometry and cell sorting

To analyze cell cycle and ploidy status of 3T3-ind-IER2 #11, B16-ind-IER2 #51, B16-ind-IER2-mNLS #56 cells, and Ret-ind-IER2 #69 cells, 1.2 × 10^5^ cells were seeded into 6-well plates and treated with three doses of DMSO or 0.2–5 μM RSL every 24 h. Cells were detached with PBS/5 mM EDTA (5–10 min/37 °C), resuspended in PBS with 1 mM MgCl_2,_ and centrifuged for 3 min at 1200 rpm. Pelleted cells were resuspended in 150 μl of 10% FBS/PBS and mixed drop-wise with ice-cold 70% EtOH. After overnight incubation at −20 °C, cells were centrifuged for 5 min at 1500 rpm, resuspended in 250 μl PBS containing 1 μl DRAQ5 fluorescent probe (5 mM; Thermo Fisher Scientific, Waltham, MA, USA) and incubated for 20 min at RT. Cells were diluted 1:1 with PBS and subjected to flow cytometry analysis using a BD FACSCanto II (BD Biosciences, Franklin Lakes, NJ, USA). Doublets and dead cells were excluded from the analysis. Flow cytometry data were analyzed using FlowJo 7.6.5 cytometry analysis software (FlowJo, LLC).

To enrich the senescent fraction in IER2-expressing B16 and Ret cells, cells were treated with 3 × 0.5 and 5 μM RSL, respectively. Six hours after the last RSL treatment, senescent and non-senescent cells were sorted using a BD FACSARIA IIu based on forward and sideward scattering, collected in RSL-containing PBS, and immediately processed for qRT-PCR or western blot analysis. In experiments in which cells were treated with either DMSO or RSL also after the cell sorting, 1 × 10^5^ sorted cells were seeded onto a 12-well plate, left to attach overnight, then treated with RSL or DMSO for additional 3 days, and subsequently processed for SA-β-galactosidase staining. After each sorting, the viability and purity of the sorted fractions were evaluated.

### Senescence-associated β-galactosidase activity assay

Senescence-associated β-galactosidase (SA-β-gal) activity was detected as previously described [34]. Briefly, 1 × 10^4^ cells/cm^2^ were seeded in triplicate into 12- to 24-well plates. For knockdown experiments, the cells were transfected immediately with siRNA. After overnight attachment, cells were treated daily with either DMSO or 0.25–5.0 μM RSL. Six hours after the last dose, cells were washed with PBS, fixed for 15 min at RT with a 2% formaldehyde/0.2% glutaraldehyde solution, then incubated overnight at 37 °C in a non-CO_2_ atmosphere with X-Gal staining solution (40 mM citric acid pH 6.0, 5 mM potassium hexacyanoferrate II, 5 mM potassium hexacyanoferrate III, 150 mM NaCl, and 2 mM MgCl_2_) containing 1 mg/ml X-β-gal substrate (Carl Roth, Karlsruhe, Germany). Cells were extensively washed with PBS and imaged with a Leica DMI6000B inverted microscope equipped with a DCF420 C camera and LAS X Core software (Leica Microsystems, Wetzlar, Germany). The percentage of SA-β-gal-positive cells from total cells was calculated for three biological replicates. Images were captured using a Zeiss Axio Imager Z1 microscope equipped with an AxioCam HRc camera and AxioVision 4.8 software (Carl Zeiss, Oberkochen, Germany).

### Proliferation assays

Proliferation assays were performed using the CyQUANT NF Cell Proliferation Assay Kit (Molecular Probes, Invitrogen/Thermo Fisher Scientific, Waltham, MA, USA). Cells were seeded in a 96-well plate at a density of 1 × 10^3^ cells/well in four biological replicates and treated daily with RSL or an equivalent volume of DMSO for two days. Cell density was measured 4 h post-seeding (effective “time 0”), as well as 24 and 48 h after the first measurement. The assay was performed according to the manufacturer’s instructions. Measurement of fluorescence was detected at 530 nm using the Infinite 200 plate reader (Tecan Group Ltd., Zürich, Switzerland). Data were calculated as the average fold increase in fluorescence at 24 and 48 h, normalized to fluorescence at 0 h.

### Cell viability assay

To determine cell viability after IER2 induction, 3T3-ind-IER2 #11 cells were treated daily with 0.5 μM RSL or an equal volume of DMSO for three consecutive days. Cells were detached 24 h after the last dose using 0.05% Trypsin–EDTA (Gibco, Carlsbad, CA, USA), resuspended in the growth medium, stained 1:1 with 0.4% Trypan Blue solution (Merck, Darmstadt, Germany) and counted with a hemocytometer.

### Migration assay

Cell exclusion zone migration assays were performed in four biological replicates using two-well silicone inserts (Ibidi, Martinsried, Germany). Cell suspension, 4 × 10^5^ cells/ml in 70 μl, were added to each well of the insert and allowed to grow to confluence overnight. Inserts were removed and images captured 0, 8, 24, and 30 h after removal. Cells were treated with RSL or DMSO at 0, 8, 24 h post-insert removal.

To assess migration of B16–F10 cells or melanocytes in the presence of conditioned media, B16 IER2 mNLS-inducible cells or IER2-wt-inducible cells with or without the expression of osteopontin were seeded around the inserts 36 h before removal of the inserts, and subsequently treated twice per day with RSL or the equivalent volume of DMSO. After 24 h, 70 µl of a 4 × 10^5^ cells/ml suspension of B16–F10 parental (non-inducible), Melan-a or Mel-STV cells were added to each well of the insert and allowed to grow to confluence overnight. Inserts were removed. Images were captured 0 h and 7–32 h after insert removal at 10x magnification using a Leica DMI6000B inverted microscope equipped with a DCF420 C camera and LAS X Core software (Leica Microsystems, Wetzlar, Germany). Wells was marked to ensure that the same area was imaged at each time point. Images were analyzed using the Fiji/ImageJ software [100], and the wound area was determined with the MRI Wound Healing Tool macro (http://dev.mri.cnrs.fr/projects/imagej-macros/wiki/Wound_Healing_Tool). The migrated area was calculated as the change in wound area.

### Transwell invasion assays

Invasion assays were performed in triplicate using Corning Costar Transwell cell culture inserts with 8 µM pores (Sigma-Aldrich/Merck, Darmstadt, Germany). Inserts were coated with 55 µl of 0.6–0.8 mg/ml High Concentration Corning Matrigel Matrix (Corning, NY, USA), which was allowed to solidify for 4 h at 37 °C. Each insert was seeded with 6 × 10^4^ cells in a medium containing 1% FBS, then were allowed to invade toward a medium containing 10% FBS for 64 h. Cells were treated with 3 × 0.5 μM (IER2-wt and OPN-KOs) or 30 μM (IER2-mNLS) RSL or an equivalent volume of DMSO twice per day. For invasion assays with conditioned media, CM was prepared by growing cells in a medium containing 1% FBS for 48 h, and treating the cells with 3 × 0.5 μM (IER2-wt and OPN-KOs) or 30 μM (IER2-mNLS) RSL, or an equivalent volume of DMSO. Transwell inserts were coated as mentioned above. Twenty-four-hour prior to seeding cells in transwell inserts, 1 × 10^5^ inducible cells were seeded in a medium containing 10% FBS to the base of the well and treated with RSL or DMSO once per day. Non-inducible B16, Melan-a or Mel-STV cells (6 × 10^4^) were seeded onto the transwell membrane in CM with 1% FBS (Melan-a, Mel-STV cells) or a mixture of CM and fresh medium containing 1% FBS (1:1, B16 cells) and allowed to invade across the membrane for 48 h.

After the invasion, cells were either fixed with methanol and stained with 0.1 mg/ml crystal violet or fixed with 70% ethanol and stained with DAPI (5 μg/ml). Inserts stained with crystal violet were incubated in 10% acetic acid to extract the crystal violet stain, and optical density was measured at 595 nm. The degree of invasion was calculated as the average fold difference in optical density or normalized DAPI-stained area of RSL treated samples compared to DMSO treated samples.

### Soft-agar assay

For the soft-agar assay, 1.5 ml of 0.5% Noble Agar (BD Biosciences, Franklin Lakes, NJ, USA) in DMEM were plated into 6-well plates and allowed to solidify at RT for at least 5 min. Soft agar at a concentration of 0.3–0.35% was prepared by dilution of 2% Noble Agar in conditioned DMEM medium of B16 IER2-ind #51 cells treated daily for three consecutive days with either 0.5 μM RSL or an equivalent volume of DMSO. Two millilitre of this dilution containing 1000–5000 cells/ml was layered on top of the bottom agar. Cells were fed twice per week with 500 μl CM and allowed to grow for 2–3 weeks. Colonies were fixed and stained with 0.005% crystal violet, 1% PFA, and 1% methanol for 1 h, imaged using a Fusion FX Imager (Vilber, Marne-la-Vallée, France), and quantified using ImageJ/Fiji [100].

### Clinical data set analysis

The cBioportal for Cancer Genomics (http://www.cbioportal.org/ [103] was used to access and download the TCGA Skin Cutaneous Melanoma Dataset containing RNA-Seq expression and clinical data of 471 melanoma patients (TCGA, provisional). The cutoff between IER2^high^ and IER2^low^ tumors was set at a *z*-score of 1.5. The difference between Kaplan–Meier curves was analyzed using the log-rank (Mantel–Cox) test. TP53, BRAF, and NRAS mutational data from whole-exome sequencing from 362 patients were correlated with IER2 expression. Significance was determined using the unpaired Student’s *t* test.

### Gene expression analysis

RNA was isolated with RNAPure, peqGOLD (Peqlab, Erlangen, Germany). RNA was tested by capillary electrophoresis on an Agilent 2100 Bioanalyzer (Agilent Technologies, Santa Clara, CA, USA) and high quality was confirmed. Gene expression profiling was performed using arrays of mouse Mm430_2.0-type from Affymetrix (Thermo Fisher Scientific, Waltham, MA, USA). Biotinylated antisense cRNA was then prepared from 33 ng/µl RNA according to the Affymetrix standard labeling protocol with the GeneChip WT Plus Reagent Kit and the GeneChip Hybridization, Wash and Stain Kit (both from Affymetrix, Santa Clara, USA). Afterward, the hybridization on the chip was performed on a GeneChip Hybridization oven 640, then dyed in the GeneChip Fluidics Station 450 and thereafter scanned with a GeneChip Scanner 3000. All of the equipment used was from Affymetrix (High Wycombe, UK). A Custom CDF Version 10 with ENTREZ-based gene definitions was used to annotate the arrays [104]. The raw fluorescence intensity values were normalized by applying quantile normalization and RMA background correction. One-way ANOVA was performed to identify differentially expressed genes using the commercial software package SAS JMP10 Genomics, version 4, from SAS (SAS Institute, Cary, NC, USA). A nominal *p*-value was taken as the level of significance. GSEA was used to determine whether defined lists (or sets) of genes exhibit a statistically significant bias in their distribution within a ranked gene list using the software GSEA [35]. Pathways belonging to various cell functions were obtained from public external databases (KEGG, http://www.genome.jp/kegg; Gene Ontology Consortium, biological processes annotations, http://www.geneontology.org/). All analyses were done using three biological replicates. The microarray data have been deposited in Gene Expression Omnibus (GEO) with accession number GSE181200.

### Statistics

Where appropriate, figure legends define the number of biological replicates (*n*). Results are represented as the mean + SEM (standard error of the mean). For each in vitro experiment, *n* represents the number of individual biological replicates. Graphs for qRT-PCR experiments represent data from *n* ≥ 3 biological replicates, with each biological replicate being executed in two technical replicates. For TCGA Skin Cutaneous Melanoma Dataset analysis, *n* = number of individual patients. For melanoma TMAs analysis, *n* = number of analyzed specimens. For experiments comparing two groups, the significance was evaluated using a two-tailed unpaired Student’s *t* test. Asterisks correspond to *p*-values below the following thresholds: *p < 0.05; **p < 0.01; ***p < 0.001; n.s. (non-significant) denotes cases where the significance threshold was not reached (*p* > 0.05). For Kaplan Meier survival analysis, significant differences were determined using the log-rank (Mantel–Cox) test. Linear regression (*R*^2^) analysis was used to assess the correlation between IER2 and OPN expression in clinical samples. Sample sizes were chosen based on previous experience with analogous experiments. Randomization was not applied. All statistics were performed with Microsoft Excel 2010 (Microsoft, Redmond, WA, USA) or GraphPad Prism 7 (GraphPad Software, La Jolla, CA, USA). Microscopy images related to in vitro experiments are representative of three biological replicates.

### Study approval

This study was performed in accordance with the ethical votes of the Medical Ethics Committee of the Medical Faculty Mannheim, University of Heidelberg (permit numbers: 2010-318N-MA and 2014-835R-MA). The study followed the guidelines of the Declaration of Helsinki, and patient identity and pathological specimens remained anonymous in the context of the study.

Human melanoma TMA was generated at the core facility of the National Center for Tumor Diseases, Department of Pathology, University of Heidelberg, as described previously [105, 106]. Information about the specimens and patients contributing to the TMA can be found in Tables S4 and S5.

## Supplementary information

Supplementary Information

Ectopic expression of IER2 in mouse 3T3-IER2-H2B-GFP fibroblasts causes morphological changes and cell division defects

Supplementary Table S1

## Data Availability

To determine phenotype-specific IER2 gene expression in human melanoma cell lines (Table S2, related to Fig. S2), the “Wagner dataset” (Gene Expression Omnibus accession number GSE8332) and the Heuristic Online Phenotype Prediction (HOPP) algorithm were used [43, 44]. All other data and/or materials are available from the corresponding author upon request. This work was supported by grants to J.P.S. and J.U. from the Deutsche Forschungsgemeinschaft (DFG, German Research Foundation)—Project number 259332240/RTG 2099, and from the European Union (HEALTH-F2-2008-201662) under the auspices of the FP7 collaborative project TuMIC.
