# Supplementary material for: IER2-induced senescence drives melanoma invasion through osteopontin
Source: Oncogene. 2021 Oct 5;40(47):6494–512. doi: 10.1038/s41388-021-02027-6 (PMC8616759; doi:10.1038/s41388-021-02027-6)
Supplement: Supplementary file 1 — Supplementary Information [file 41388_2021_2027_MOESM1_ESM.pdf]

Supplementary information

IER2-induced senescence drives melanoma invasion through osteopontin

by Kyjacova et al.

Supplementary Figures

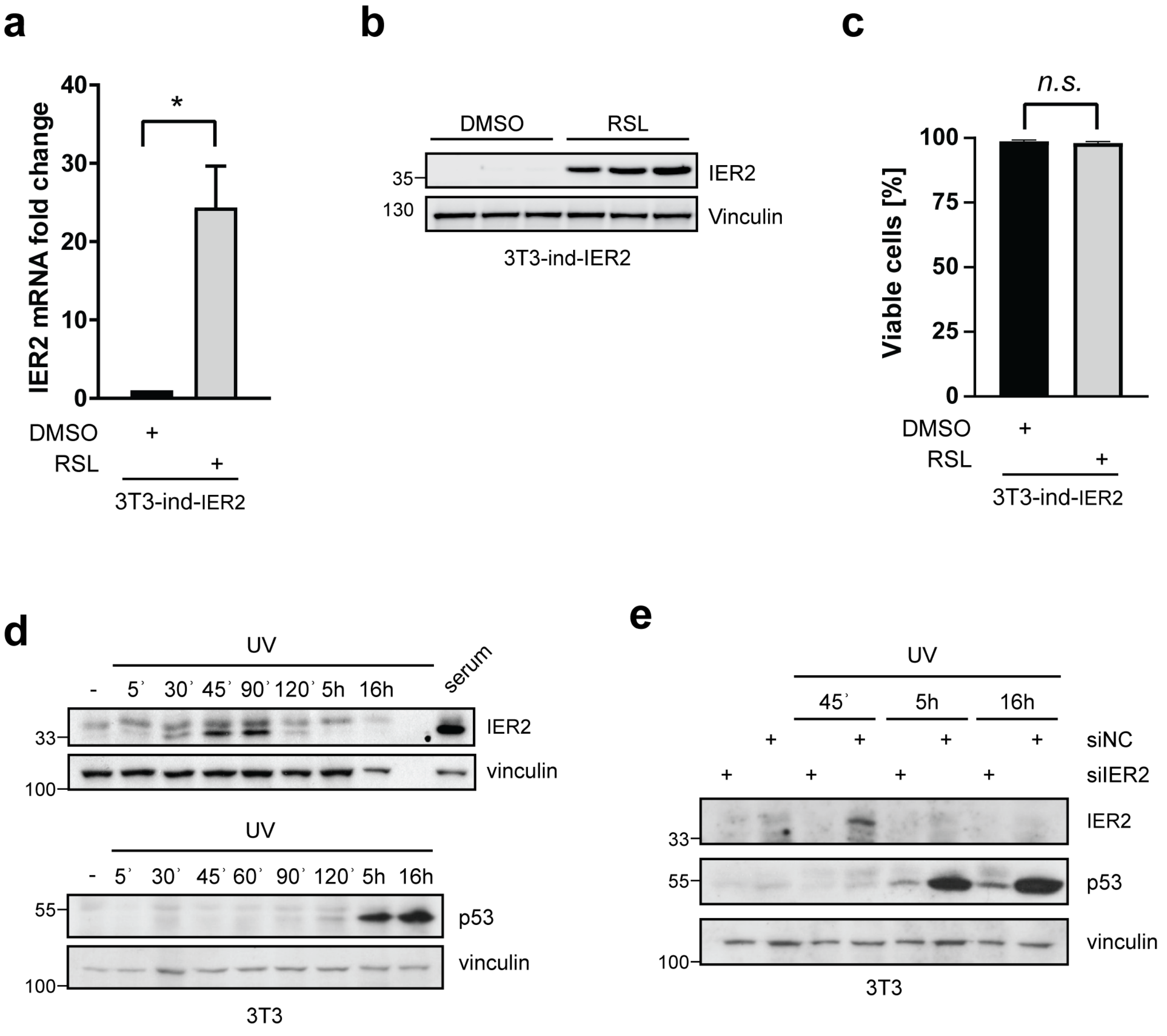

Figure S1 | IER2 induction using RheoSwitch-regulated gene expression and after UV irradiation. **a** Real time qRT-PCR quantification of IER2 in 3T3-ind-IER2 #11 cells treated with either DMSO or RSL (3 x 0.5 µM). RPLP0 was used as a reference gene. Bars represent mean + SEM from 3 biological replicates analyzed in 2 technical duplicates. *$p<0.05$. Significance was determined using an unpaired Student's t-test. **b** Western blot analysis of IER2 and vinculin (loading control) in 3T3-ind-IER2 #11 cells treated with either DMSO or RSL for 3 days (3 x 0.5 µM). Three separate biological replicates are shown

for each condition. **c** Quantification of viable 3T3-ind-IER2 #11 cells after 3 days of treatment with either DMSO or RSL (3 x 0.5 µM). Bars represent mean + SEM; n=3. **d** Western blot analysis of IER2, p53 and vinculin (loading control) in 3T3 cells irradiated with UV-light (254 nm, 30 J/m^2). Cells were harvested at the indicated time points. Pre-starved 3T3 cells (48 h in DMEM/0.6% FCS) stimulated 60 min with DMEM/20% FCS served as a control for IER2 expression ("serum"). **e** Western blot analysis of IER2, p53, and vinculin in 3T3 cells irradiated with UV-light (254 nm, 30 J/m^2) with (siIER2) or without (siNC) siRNA-mediated IER2 knockdown. Cells were harvested at the indicated time points. n.s., non-significant ($p>0.05$); *$p<0.05$. Significance was determined using an unpaired Student's t-test.

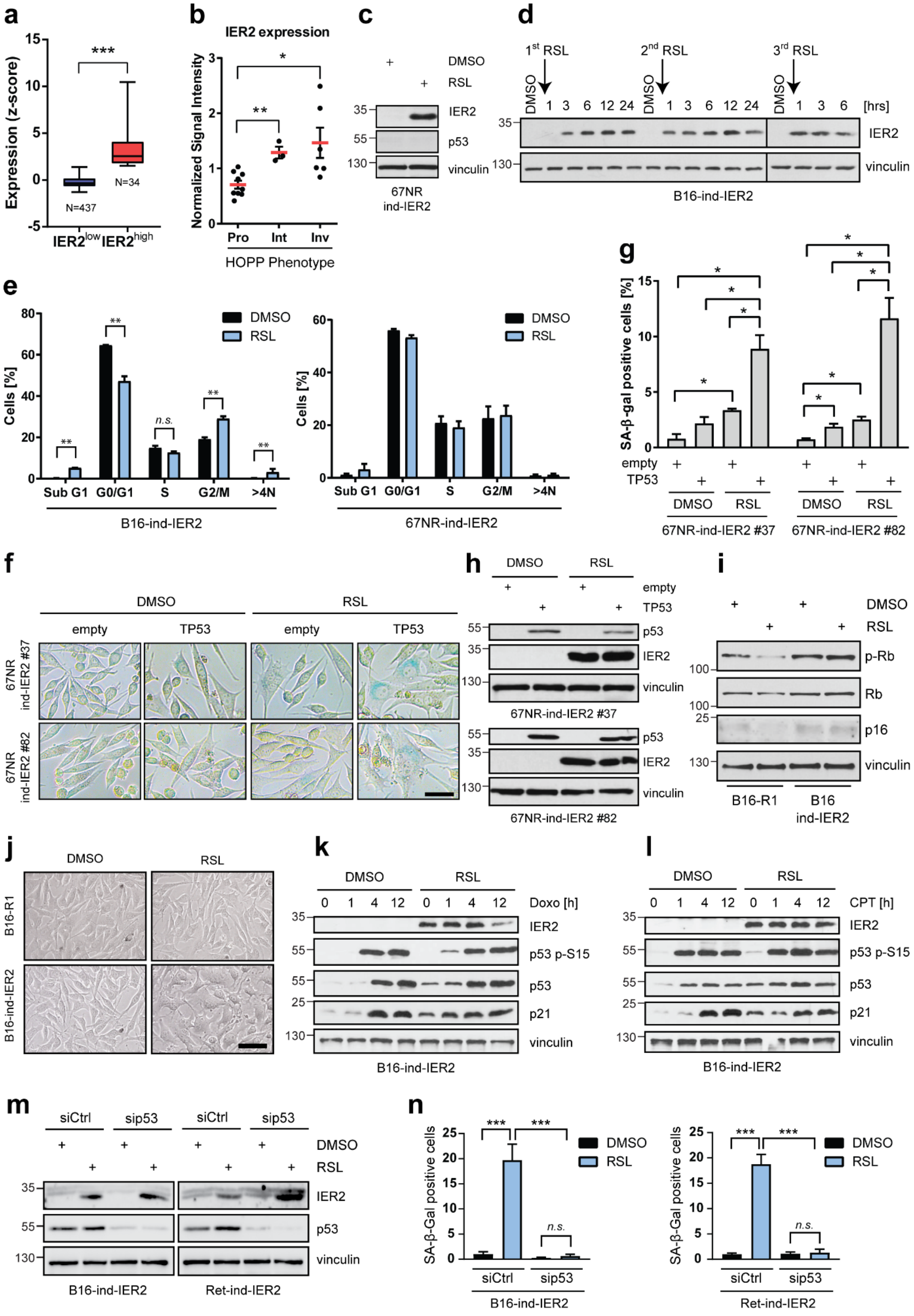

Figure S2 | IER2 promotes the induction of senescence in a p53-dependent manner. a TCGA skin

cutaneous melanoma cohort stratified on the basis of IER2 expression into samples with low ($IER2^{low}$) and high ($IER2^{high}$) IER2 levels. Solid lines inside the boxes show the medians; the box limits extend from the 25th to 75th percentiles, whiskers indicate minimum to maximum values. **b** Proliferative (Pro), intermediate (Int), and invasive (Inv) phenotype-specific IER2 gene expression in human melanoma cell lines (see **Table S2**) in the "Wagner dataset" (Gene Expression Omnibus accession number: GSE8332) based on the Heuristic Online Phenotype Prediction algorithm (HOPP; [1, 2]). Red solid lines in the dot plots represent means, and the error bars represent + SEM. **c** Western blot analysis of IER2, p53, and vinculin (loading control) in 67NR-ind-IER2 #37 cells after treatment with either DMSO or RSL (3 x 0.5 μM). **d** Western blot analysis of IER2 and vinculin in B16-ind-IER2 #51 cells treated daily with RSL (0.5 μM) without change of medium. Cells were harvested in indicated times. DMSO-treated cells served as a control. **e** Cell cycle distribution of B16-ind-IER2 #51 (left) and 67NR-ind-IER2 #37 (right) cells measured by flow cytometry after treatment with DMSO or RSL (3 x 0.5 μM). Bars represent mean + SEM; n=3; 20 000 cells per replicate were analyzed. **f, g** Representative images showing SA-β-galactosidase (SA-β-gal)-positive cells (blue; **f**) and quantification of SA-β-gal-positive cells **g** in 67NR-ind-IER2 #37 and 67NR-ind-IER2 #82 cells transfected with pcDNA3.1-empty (empty) or pcDNA3.1-TP53 (TP53) plasmid, and treated with either DMSO or RSL (3 x 5 μM). Bars represent means + SEM of 3 biological replicates. *$p<0.05$. Statistical significance was determined using an unpaired Student's t-test. Scale bar, 50 μm. **h** Western blot analysis of p53, IER2, and vinculin in 67NR-ind-IER2 #37 and 67NR-ind-IER2 #82 cells transfected with pcDNA3.1-empty (empty) or pcDNA3.1-TP53 (TP53) plasmid followed by treatment with DMSO or RSL (3 x 5 μM). **i** Western blot analysis of Rb phosphorylated on serine 807/811 (p-S807/811), Rb, p16, and vinculin in parental B16-R1 #2 and B16-ind-IER2 #51 cells after treatment with either DMSO or RSL (3 x 0.5 μM). **j** Representative microscopy images of parental B16-R1 #2 and B16-ind-IER2 #51 cells treated with either DMSO or RSL (3 x 0.5 μM). Scale bar, 50 μm. **k, l** Western blot analysis of IER2, p53 p-S15, p53, p21, and vinculin in B16-ind-IER2 #51 cells treated with doxorubicin (Doxo, 2 μM; **k**) or camptothecin (CPT, 2 μM; **l**) for the indicated times in the presence or absence of RSL (3 x 0.5 μM). DMSO was used as control. **m** Western blot analysis of IER2, p53, and vinculin (loading control) in B16-ind-IER2 #51 and Ret-ind-IER2 #69 cells that were transfected with small interfering RNA against p53 (sip53) or non-silencing control (siCtrl) and treated with either DMSO or RSL (3 x 0.5 μM). **n** Quantification of SA-β-gal-positive B16-ind-IER2 #51 and Ret-ind-IER2 #69 cells that were transfected with small interfering RNA against p53 (sip53) or non-silencing control (siCtrl) and treated with either DMSO or RSL (3 x 0.5 μM). The data were normalized to the siCtrl DMSO condition and analyzed using one-way ANOVA with Sidak's multiple comparisons test. n.s., non-significant ($p>0.05$), ***$p<0.001$.

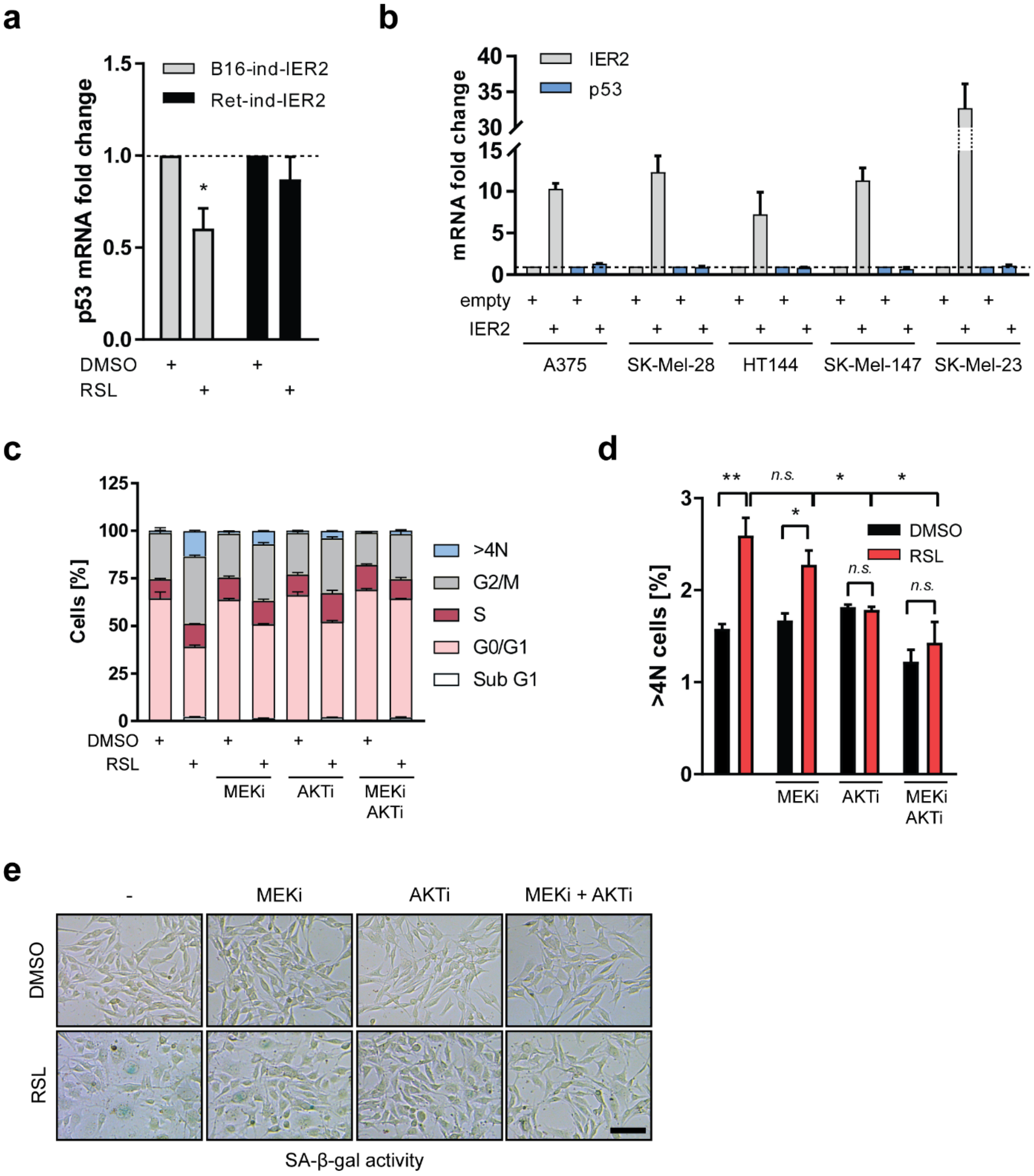

Figure S3 | IER2 requires active AKT and ERK signaling to promote cell cycle alterations in murine melanoma cells. a Real time qRT-PCR quantification of p53 expression in B16-ind-IER2 #51 and Ret-ind-IER2 #69 cells treated with either DMSO or RSL (3 x 0.5 µM). RPLP0 was used for normalization. Bars represent means + SEM; n=3. **b** Real time qRT-PCR quantification of IER2 and p53 expression in A375, SK-Mel-28, HT144, SK-Mel-147, and SK-Mel-23 human melanoma cells transfected either with pcDNA3.1-empty (empty) pcDNA3.1-IER2 (IER2) plasmid for 48 h. GAPDH was used for normalization. Bars represent means + SEM; n=3 (A375, SK-Mel-28) or 2 (HT144, SK-Mel-147, SK-Mel-23). **c** DRAQ5 labeling of nuclear DNA followed by flow cytometry quantification of B16-ind-IER2 #51 cells treated with DMSO or with RSL (3 x 0.5 µM) in combination with MEKi (3 x 1 µM)

and/or AKTi (3 x 1 µM). Bars represent means + SEM; n=3. **d** DRAQ5 labeling of nuclear DNA followed by flow cytometry quantification of >4N Ret-ind-IER2 #69 cells treated either with DMSO or with RSL (3 x 0.5 µM) in combination with MEKi (3 x 1 µM) and/or AKTi (3 x 1 µM). Bars represent means + SEM; n=3. n.s., non-significant (p>0.05), *p<0.05, ** p<0.01. Statistical significance was determined using an unpaired Student's t-test. **e** Staining for SA-β-galactosidase activity (blue) in B16-ind-IER2 #51 cells, treated as in **c**. Scale bar, 100 µm. Quantification of the percentage of SA-β-galactosidase-positive cells is shown in Figure 3d.

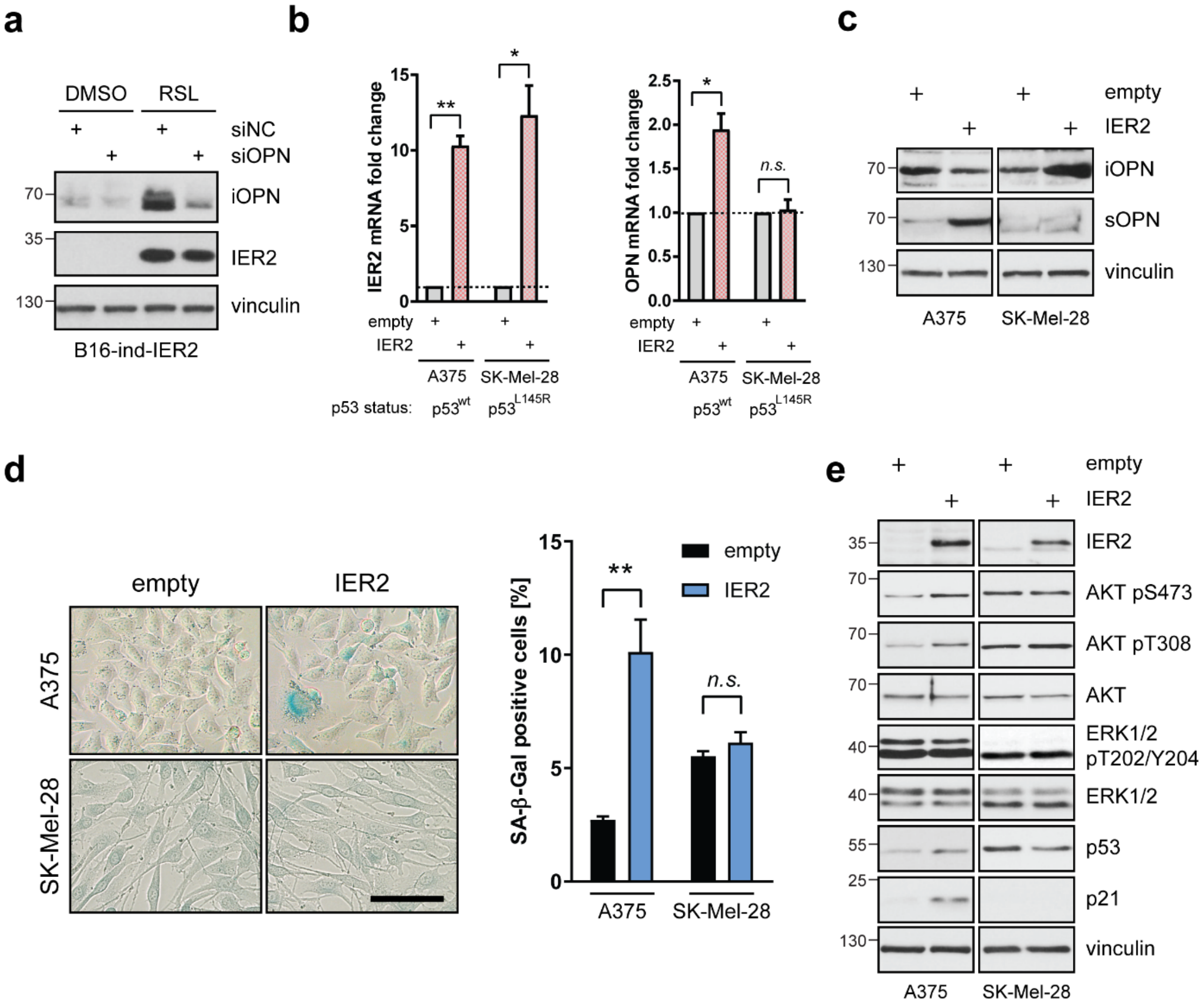

Figure S4 | Characterization of the IER2-induced SASP. a Western blot analysis of intracellular OPN (iOPN), IER2, and vinculin (loading control) in B16-ind-IER2 #51 cells treated with either DMSO or RSL (2 x 0.5 μM), with (siOPN) or without (siNC) siRNA-mediated OPN knockdown. **b** Real time qRT-PCR quantification of IER2 (left) and OPN (right) in human melanoma A375 and SK-Mel-28 cells 48 h after the transfection with pcDNA3.1 or pcDNA3.1-IER2 plasmid. GAPDH was used for normalization. Bars represent means + SEM; n=3. **c** Western blot analysis of intracellular OPN (iOPN), secreted OPN (sOPN), and vinculin (loading control) in A375 and SK-Mel-28 cells 48 h after the transfection with pcDNA3.1 or pcDNA3.1-IER2 plasmid **d** Representative images showing SA-β-galactosidase (SA-β-gal)-positive cells (blue, left) and quantification of SA-β-gal-positive A375 and SK-Mel-28 cells (right) 48 and 72 h after the transfection with pcDNA3.1 or pcDNA3.1-IER2 plasmid, respectively. Bars represent means + SEM; n=3. **e** Western blot analysis of indicated proteins in A375 and SK-Mel-28 cells treated as in C. n.s., non-significant (p>0.05), *p<0.05, ** p<0.01. Statistical significance was determined using an unpaired Student's t-test.

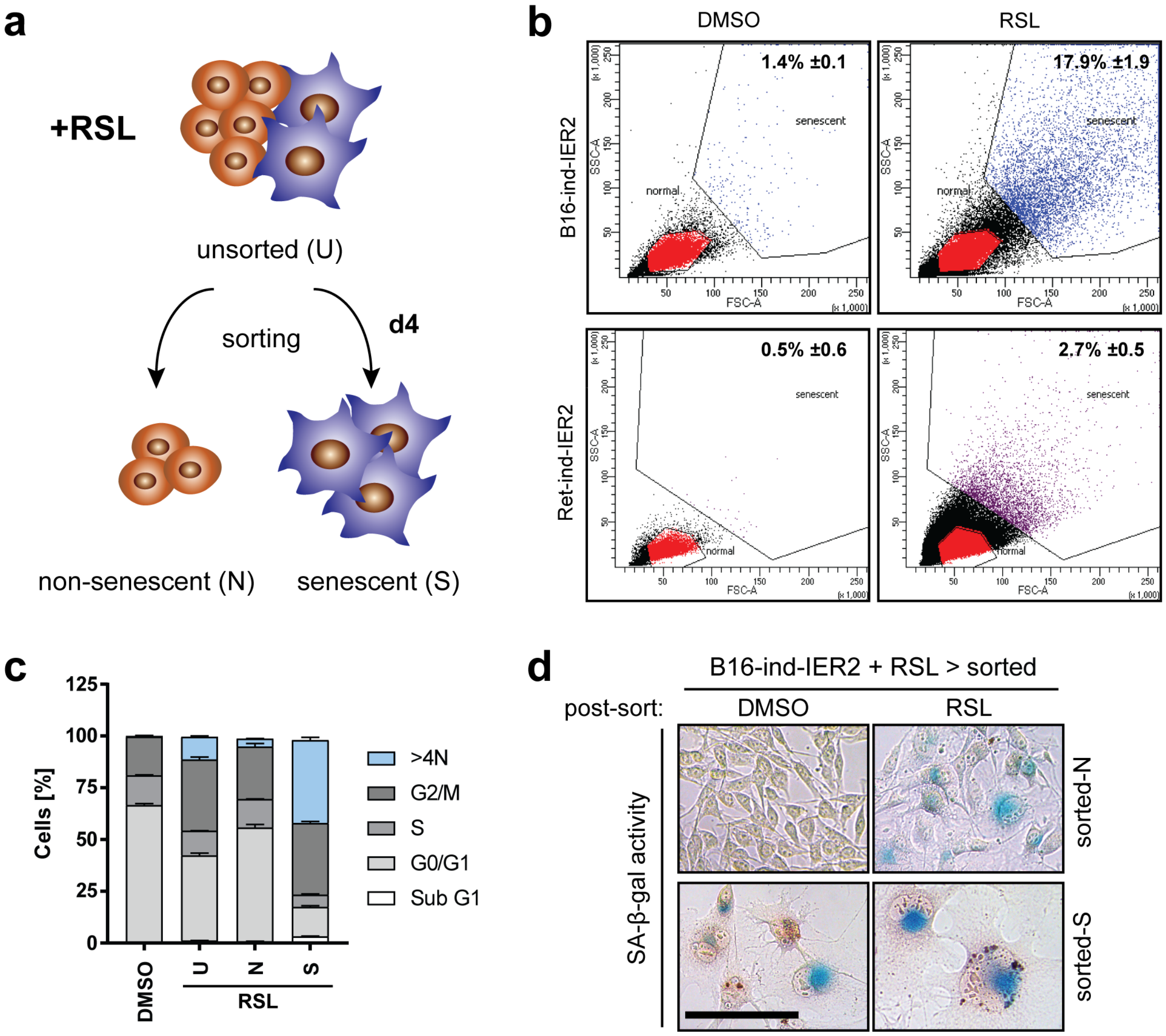

Figure S5 | Flow cytometry-based sorting of senescent-like cells and cell cycle analysis. a Schematic depiction of the sorting strategy for senescent (S) and non-senescent (N) RSL-treated B16-ind-IER2 #51 cells. **b** Flow cytometry plots showing the gating strategy to sort non-senescent from senescent B16-ind-IER2 #51 and Ret-ind-IER2 #69 cells after treatment with DMSO or RSL (3 x 0.5 μM and 3 x 1.0 μM, for the B16-ind-IER2 #51 and Ret-ind-IER2 #69 cells, respectively). **c** DRAQ5 labeling of nuclear DNA followed by flow cytometry analysis of cell cycle distribution of DMSO- and RSL-treated (3 x 0.5 μM) unsorted (U), sorted non-senescent (N) and sorted senescent (S) B16-ind-IER2 #51 cells. Bars represent means + SEM; n=3. **d** B16-ind-IER2 #51 cells were treated for 3 days (3 x 0.5 μM) with RSL and sorted into non-senescent and senescent populations, each of which was subsequently treated for 3 days with DMSO or RSL (3 x 0.5 μM). Representative images of an SA-β-gal assay are shown. Scale bar, 100 μm. Quantification of the percentage of SA-β-gal-positive cells is shown in Figure 5g.

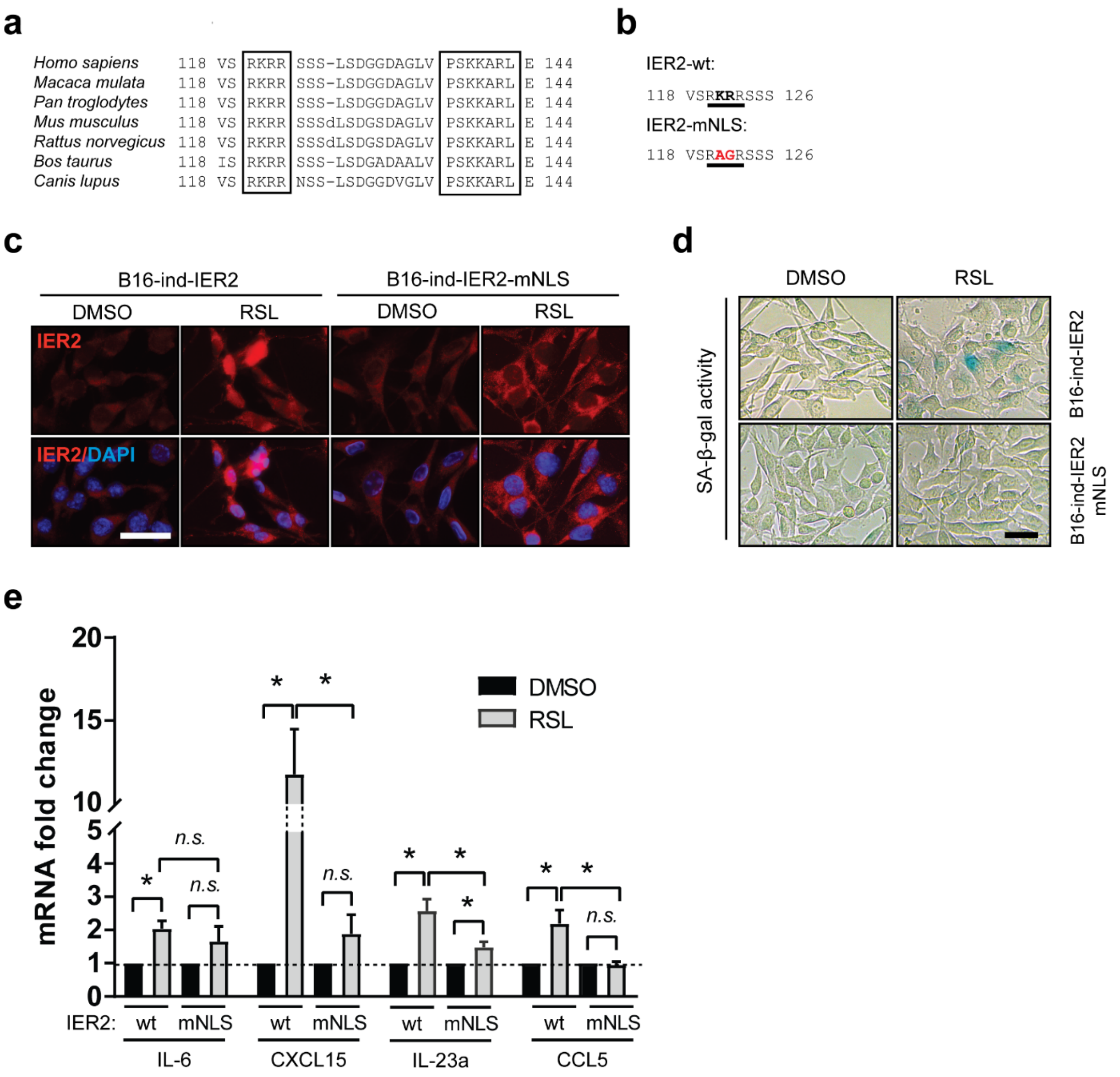

Figure S6 | The IER2 NLS mutant is localized in the cytoplasm and has an attenuated ability to induce expression of SASP genes. a Comparison of the amino acid sequences of the IER2 putative bipartite nuclear localization signal (NLS) across different species. The conserved basic amino acid consensus sequences are boxed. **b** Sequences of the wild-type IER2 NLS and the IER2 NLS mutant (IER2-mNLS). **c** Representative microscopy images illustrating the cellular localization of IER2 (red) detected by indirect immunofluorescence in B16-ind-IER2 #51 and B16-ind-IER2-mNLS #56 cells treated with either DMSO or RSL (3 x 0.25 µM and 3 x 5.0 µM RSL for the B16-ind-IER2 #51 and B16-ind-IER2-mNLS #56 cells, respectively). DAPI (1 µg/ml; blue) was used to counterstain the nuclei. Scale bar, 50 µm. **d** Representative images of SA-β-galactosidase (SA-β-gal)-positive B16-ind-IER2 #51 cells (treated with DMSO or 3 x 0.25 µM RSL) and B16-ind-IER2-mNLS #56 cells (treated with DMSO or 3 x 5 µM RSL). Scale bar, 50 µm. Quantification of the SA-β-gal staining is shown in Figure 6c. **e** Real time qRT-PCR quantification of IL-6, CXCL15, IL-23a, and CCL5 in B16-ind-IER2 #51 and B16-ind-IER2mNLS #56 cells treated with either DMSO or RSL (3 x 0.25 µM and 3 x 5.0 µM RSL for the B16-ind-IER2 #51 and B16-ind-IER2-mNLS #56 cells, respectively). RPLP0 was used for normalization. Bars

represent means + SEM; n=3 (IL-6) or 5. n.s., non-significant ($p>0.05$), *$p<0.05$. Statistical significance was determined using an unpaired Student's t-test.

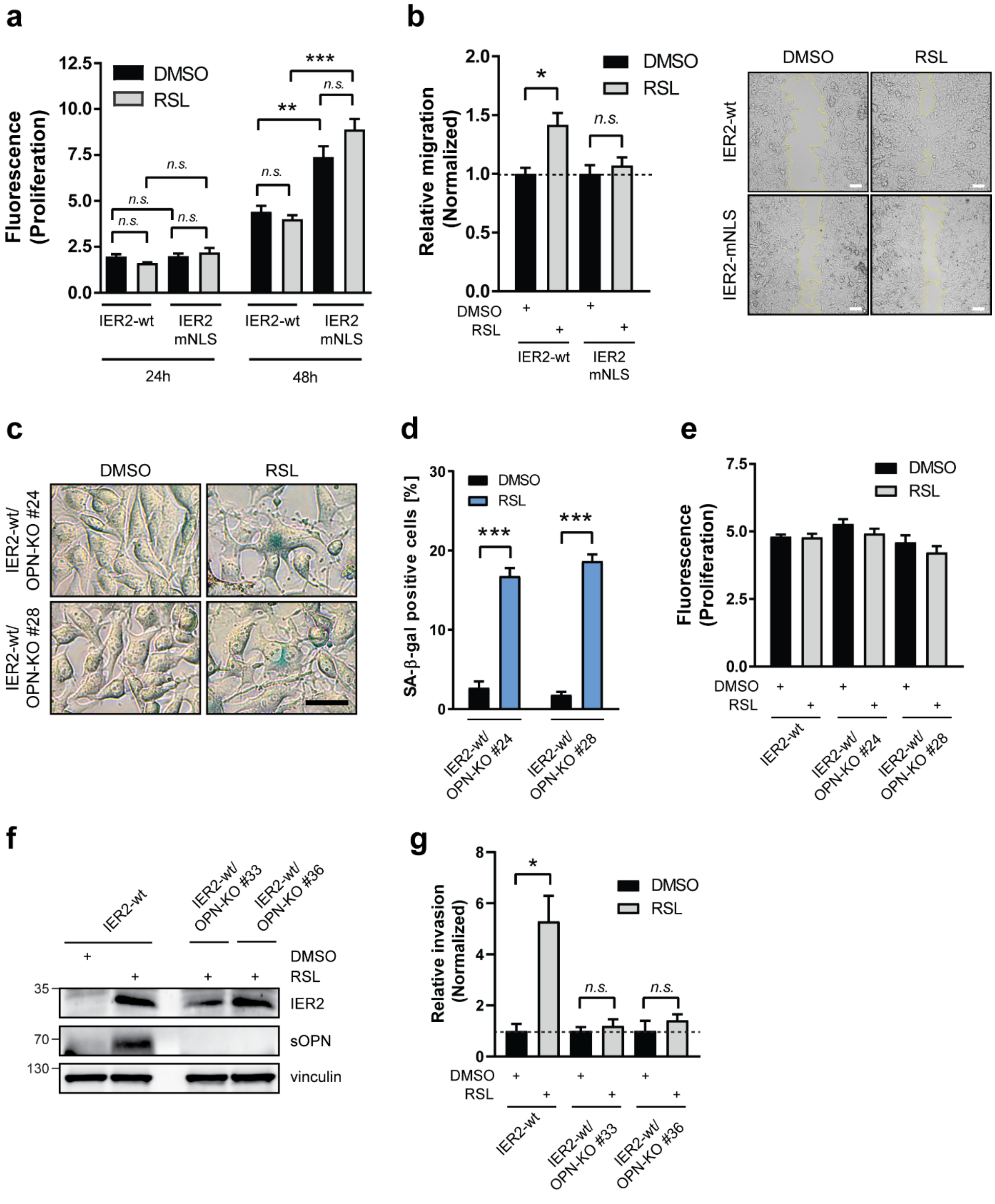

Figure S7 | Proliferation, migration and senescence of cells expressing the IER2 NLS mutant or lacking osteopontin. a CyQUANT cell proliferation assay with B16-ind-IER2 #51 (IER2-wt#51) and B16-ind-IER2-mNLS #56 (IER2-mNLS #56) cells treated with either DMSO or RSL (2 x 0.25 µM and 5 µM RSL for the B16-ind-IER2 #51 and B16-ind-IER2-mNLS #56 cells, respectively) at the indicated times. Data are expressed as fold change of fluorescence normalized to the start of the experiment. Bars represent mean + SEM from 4 biological replicates. **b** Migration of RSL-treated B16-ind-IER2 #51 (3 x 0.25 µM) and B16-ind-IER2-mNLS #56 (3 x 5 µM) cells 30 h after the removal of silicon inserts. Data are normalized to DMSO-treated cells. Bars represent means + SEM; n=3-4. Scale bar, 100 µm.

c, d Representative images showing SA-β-galactosidase (SA-β-gal)-positive cells (blue; **c**) and quantification of SA-β-gal-positive B16-ind-IER2-OPN-KO #24 (OPN-KO #24) and B16-ind-IER2-OPN-KO #28 (OPN-KO #28) cells **d** treated either with DMSO or RSL (3 x 0.5 µM). Bars represent mean + SEM from 3 biological replicates. Scale bar, 50 µm. **e** CyQUANT cell proliferation assay of B16-ind-IER2#51, B16-ind-IER2-OPN-KO #24 (OPN-KO #24), and B16-ind-IER2-OPN-KO #28 (OPN-KO #28) cells treated with either DMSO or RSL (2 x 5.0 µM) at 48 h. Data are expressed as fold change fluorescence normalized to the start of the experiment. Bars represent mean + SEM; n=4. **f** Western blot analysis of IER2, secreted OPN (sOPN) and vinculin (loading control) in B16-ind-IER2 #51 (IER2-wt) cells, as well as in B16-ind-IER2-OPN-KO #33 (IER2-wt/OPN-KO #33) and B16-ind-IER2-OPN-KO #36 (IER2-wt/OPN-KO #36) cells, in which osteopontin was deleted through CRISPR/Cas9-mediated gene inactivation, treated with DMSO or RSL (3 x 0.5 µM). **g** Invasion of B16-ind-IER2 #51, B16-ind-IER2-OPN-KO #33 and B16-ind-IER2-OPN-KO #36 cells seeded in DMEM/1% FBS and treated either with DMSO or RSL (5 x 0.5 µM). Data represent relative invasion through a Matrigel layer (0.6 mg/ml) towards DMEM/10% FBS at 64 h and are shown as mean + SEM, normalized to DMSO-treated controls; n=3. n.s., non-significant ($p>0.05$), *$p<0.05$. **$p<0.01$, ***$p<0.001$. Statistical significance was determined using an unpaired Student's t-test.

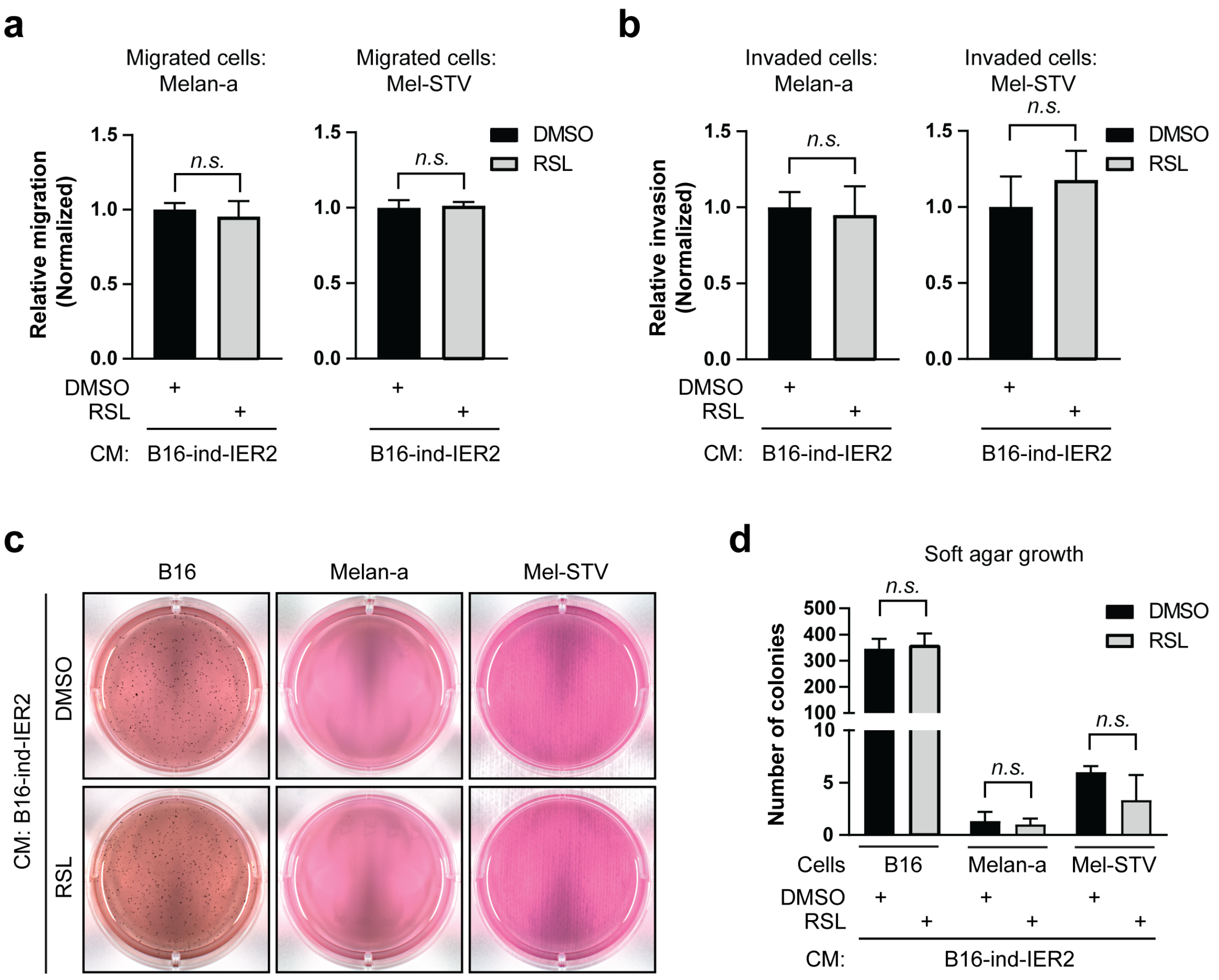

Figure S8 | Conditioned medium from IER2-expressing B16 cells, does not affect the migration, invasion or soft agar growth of normal melanocytes. a Migration of normal murine Melan-a melanocytes and human Mel-STV melanocytes in the presence of medium conditioned by B16-ind-IER2 #51 cells that were treated with DMSO or RSL (3 x 0.5 µM), 7 h after removal of silicone inserts. Data represent relative migration + SEM from 3-4 biological replicates normalized to DMSO-treated controls. **b** Invasion of normal murine Melan-a and human Mel-STV melanocytes in the presence of conditioned medium (CM) from B16-ind-IER2 #51. Data represent relative invasion through a Matrigel layer (0.6 mg/ml) towards DMEM/10% FBS at 48 h and are shown as mean + SEM; n=3. Data are normalized to DMSO-treated controls. **c, d** Murine Melan-a and human Mel-STV melanocytes were seeded in soft-agar and cultivated for two weeks in the presence of CM from B16-ind-IER2 #51 cells that were treated either with DMSO or RSL (3 x 0.5 µM). Parental B16 cells were used as a positive control for colony formation in soft agar. Representative images are shown in (**c**) and quantification of the number of colonies formed are presented in **d** (n=3). n.s., non-significant ($p>0.05$), as determined by an unpaired Student's t-test.

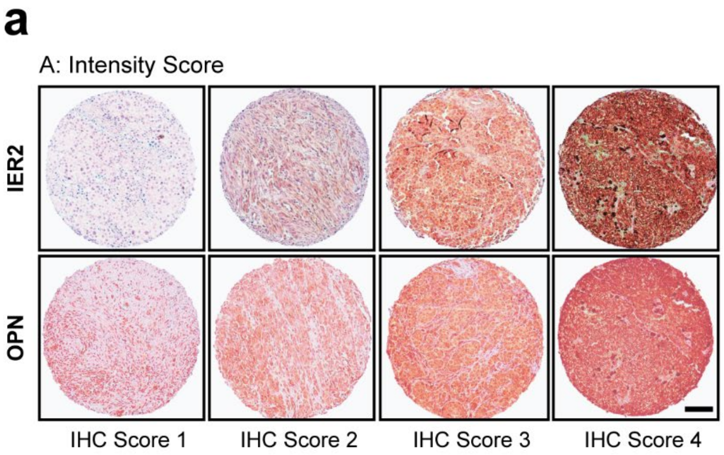

B: % of S100B-positive cells that are positive for IER2/OPN	IHC Score
0 %	0
1-25 %	1
26-50 %	2
51-75 %	3
76-100 %	4

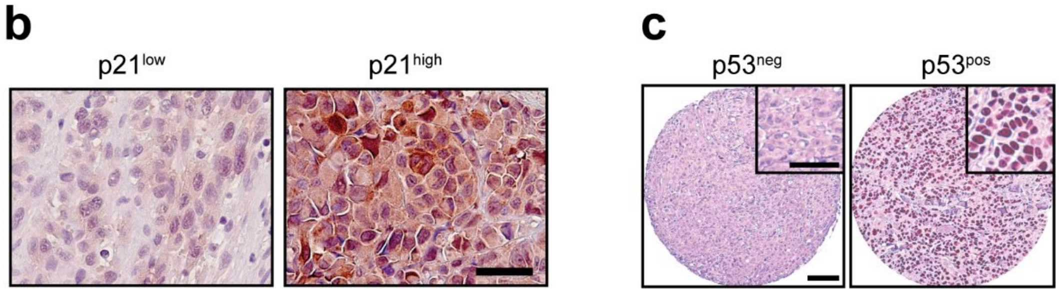

Fig S9 IER2 expression in human melanoma cells and tissue. a Representative images illustrating the IHC intensity scores of IER2- and OPN-stained TMAs (panel A) and a table summarizing the quantitative IHC scoring system (panel B). Immunohistochemistry scores were calculated by multiplying the intensity and quantity scores according to the formula: IHC Score = A x B. Scale bar, 100 μM. **b** Representative images of low ($p21^{low}$) and high ($p21^{high}$) p21 IHC staining in TMAs. Scale bar, 50 μm. **c** Representative images of nuclear p53 negative ($p53^{neg}$) and p53 nuclear positive ($p53^{pos}$) IHC staining in TMAs. Scale bars, 100 μm (punch); 50 μm (zoom).

Supplemental Tables

Table S1 Gene Set Enrichment Analysis (GSEA) of GO and KEGG gene sets in 3T3-ind-IER2 #11 cells stimulated with RSL (0.2 μM) for 24 h.

Table S2 Heuristic Online Phenotype Prediction (HOPP; Hoek et al., 2006; Widmer et al., 2012) of human melanoma cell lines and melanoma phenotype-specific IER2 gene expression in the "Wagner dataset" (Omnibus Database: GSE8332).

Melanoma Cell Line	IER2 Fold Change	HOPP Phenotype
624	0.624	Proliferative
888	0.552	Proliferative
928MEL	0.946	Proliferative
G361	0.894	Proliferative
MALME3M	0.413	Proliferative
MeWo	0.534	Proliferative
SK23	0.555	Proliferative
SKMEL28	1.028	Proliferative
A2058	0.810	Proliferative
527MEL	1.143	Intermediate
C32	1.493	Intermediate
COLO829	1.227	Intermediate
A375	2.092	Invasive
Hs294T	0.843	Invasive
Hs695T	1.000	Invasive
LOXIMVI	1.348	Invasive
MDA435	1.015	Invasive
RPMI7951	2.489	Invasive

Table S3 Fold regulation of Cancer Inflammation and Immunity Crosstalk Array genes following IER2 induction. mRNA levels of the genes included in the Cancer Inflammation and Immunity Crosstalk Array (Qiagen, #PAMM-181Z) in B16-ind-IER2 #51 cells treated with 3 doses of RSL (0.5 µM). Fold change >2.0 and *p*-values <0.05 are shown in red.

Symbol	Description	Fold Regulation[A]	*p*-value
**Ackr3**	Chemokine (C-X-C motif) receptor 7	2.0504	0.040511
**Aicda**	Activation-induced cytidine deaminase	1.222	0.54312
**Bcl2**	B-cell leukemia/lymphoma 2	1.1507	0.781521
**Bcl2l1**	Bcl2-like 1	-1.1654	0.134765
**Ccl2**	Chemokine (C-C motif) ligand 2	1.0205	0.996035
**Ccl20**	Chemokine (C-C motif) ligand 20	1.5756	0.257819
**Ccl22**	Chemokine (C-C motif) ligand 22	1.3249	0.581821
**Ccl28**	Chemokine (C-C motif) ligand 28	-1.0192	0.962331
**Ccl4**	Chemokine (C-C motif) ligand 4	1.0737	0.779371
**Ccl5**	Chemokine (C-C motif) ligand 5	4.8992	0.006726
**Ccr1**	Chemokine (C-C motif) receptor 1	-1.0724	0.873104
**Ccr10**	Chemokine (C-C motif) receptor 10	-1.0503	0.485136
**Ccr2**	Chemokine (C-C motif) receptor 2	1.0491	0.984274
**Ccr4**	Chemokine (C-C motif) receptor 4	1.1038	0.655916
**Ccr5**	Chemokine (C-C motif) receptor 5	1.3097	0.65207
**Ccr7**	Chemokine (C-C motif) receptor 7	1.2448	0.467088
**Ccr9**	Chemokine (C-C motif) receptor 9	1.0347	0.958071
**Cd274**	CD274 antigen	1.3972	0.014738
**Csf1**	Colony stimulating factor 1 (macrophage)	1.8182	0.001383
**Csf2**	Colony stimulating factor 2 (granulocyte-macrophage)	1.0887	0.260341
**Csf3**	Colony stimulating factor 3 (granulocyte)	1.0228	0.928189
**Ctla4**	Cytotoxic T-lymphocyte-associated protein 4	-1.0406	0.895733
**Cxcl1**	Chemokine (C-X-C motif) ligand 1	1.1831	0.537649
**Cxcl10**	Chemokine (C-X-C motif) ligand 10	1.9487	0.005161
**Cxcl11**	Chemokine (C-X-C motif) ligand 11	-1.0699	0.705171
**Cxcl12**	Chemokine (C-X-C motif) ligand 12	1.2476	0.586836
**Cxcl2**	Chemokine (C-X-C motif) ligand 2	-1.3021	0.346716
**Cxcl5**	Chemokine (C-X-C motif) ligand 5	1.0041	0.895982
**Cxcl9**	Chemokine (C-X-C motif) ligand 9	1.1038	0.809786
**Cxcr1**	Chemokine (C-X-C motif) receptor 1	1.0395	0.786497
**Cxcr2**	Chemokine (C-X-C motif) receptor 2	1.1695	0.603924
**Cxcr3**	Chemokine (C-X-C motif) receptor 3	1.5683	0.223015
**Cxcr4**	Chemokine (C-X-C motif) receptor 4	1.0861	0.683165
**Cxcr5**	Chemokine (C-X-C motif) receptor 5	1.2946	0.285025
**Egf**	Epidermal growth factor	1.1481	0.737226
**Egfr**	Epidermal growth factor receptor	1.0786	0.800717
**Fasl**	Fas ligand (TNF superfamily, member 6)	1.3403	0.736124
**Foxp3**	Forkhead box P3	1.0064	0.893575
**Gbp2b**	Guanylate binding protein 1	1.7891	0.015386
**Gzma**	Granzyme A	1.0912	0.724854
**Gzmb**	Granzyme B	1.42	0.336008
**H2-D1**	Histocompatibility 2, D region locus 1	2.4723	0.000023
**H2-K1**	Histocompatibility 2, K1, K region	2.0409	0.000045
**Hif1a**	Hypoxia inducible factor 1, alpha subunit	1.3341	0.020632
**Ido1**	Indoleamine 2,3-dioxygenase 1	1.0887	0.827916
**Ifng**	Interferon gamma	1.0064	0.956425
**Igf1**	Insulin-like growth factor 1	1.2798	0.446747

**Il10**	Interleukin 10	1.0516	0.967086
**Il12a**	Interleukin 12A	1.1244	0.715431
**Il12b**	Interleukin 12B	1.2362	0.721096
**Il13**	Interleukin 13	1.0181	0.963095
**Il15**	Interleukin 15	1.8351	0.003286
**Il17a**	Interleukin 17A	-1.2037	0.602958
**Il1a**	Interleukin 1 alpha	-1.0192	0.942483
**Il1b**	Interleukin 1 beta	1.109	0.81995
**Il1r1**	Interleukin 1 receptor, type I	1.2107	0.745509
**Il2**	Interleukin 2	-1.0382	0.929979
**Il22**	Interleukin 22	1.0276	0.978072
**Il23a**	Interleukin 23, alpha subunit p19	2.3175	0.009864
**Il4**	Interleukin 4	1.0836	0.783191
**Il5**	Interleukin 5	1.1913	0.572609
**Il6**	Interleukin 6	1.9759	0.129744
**Irf1**	Interferon regulatory factor 1	1.0299	0.617761
**Kitl**	Kit ligand	-1.0848	0.505324
**Mif**	Macrophage migration inhibitory factor	1.2079	0.008211
**Myc**	Myelocytomatosis oncogene	1.0252	0.972774
**Myd88**	Myeloid differentiation primary response gene 88	2.3175	0.000324
**Nfkb1**	Nuclear factor of kappa light polypeptide gene enhancer in B-cells 1, p105	-1.4548	0.001198
**Nos2**	Nitric oxide synthase 2, inducible	-1.2812	0.470453
**Pdcd1**	Programmed cell death 1	1.0443	0.844643
**Ptgs2**	Prostaglandin-endoperoxide synthase 2	-1.0263	0.987313
**Spp1 (OPN)**	Secreted phosphoprotein 1	22.3553	0.000318
**Stat1**	Signal transducer and activator of transcription 1	1.9487	0.00066
**Stat3**	Signal transducer and activator of transcription 3	1.3876	0.190671
**Tgfb1**	Transforming growth factor, beta 1	1.5539	0.043534
**Tlr2**	Toll-like receptor 2	3.0228	0.00499
**Tlr3**	Toll-like receptor 3	1.3684	0.007817
**Tlr4**	Toll-like receptor 4	1.6387	0.004217
**Tlr7**	Toll-like receptor 7	1.268	0.263599
**Tlr9**	Toll-like receptor 9	1.0323	0.931722
**Tnf**	Tumor necrosis factor	1.1454	0.627715
**Tnfsf10**	Tumor necrosis factor (ligand) superfamily, member 10	1.0589	0.846674
**Trp53**	Transformation related protein 53	-1.1051	0.480875
**Vegfa**	Vascular endothelial growth factor A	1.2362	0.124654

[A] Fold Regulation: treated cells vs. DMSO-treated cells.

Table S4 Description of tissue microarray specimens used for IER2 and OPN expression and correlation analysis.

Parameter		IER2 Expression Graph		OPN Expression Graph		IER2/OPN Correlation Analysis	
**TMAs characteristics**							
**Total no. of specimens**		*n*		*n*		*n*	
		121		130		120	
Specimen type		*n*	%	*n*	%	*n*	%
MN		19	15.7	19	14.6	19	15.8
PT		46	38.0	48	36.9	45	37.5
DM		56	46.2	63	48.5	56	46.7
**Total no. of patients**		*n*		*n*		*n*	
		104		111		103	
Specimen type		*n*	%	*n*	%	*n*	%
MN		18	17.3	18	16.2	18	17.5
PT		45	43.3	47	42.4	44	42.7
DM		41	39.4	46	41.4	41	39.8
**Clinical Characteristics**							
**Sex**		*n*	%	*n*	%	*n*	%
MN	Female	9	50.0	9	50.0	9	50.0
	Male	9	50.0	9	50.0	9	50.0
PT	Female	9	20.0	11	23.4	9	20.5
	Male	36	80.0	36	76.6	35	79.5
DM	Female	18	43.9	18	39.1	18	43.9
	Male	23	56.1	28	60.9	23	56.1
**Age at Diagnosis**		Mean in years (range)					
MN		59.8 (39-81)		59.8 (39-81)		59.8 (39-81)	
PT		63.3 (34-91)		63.6 (34-91)		63.7 (34-91)	
DM		65.0 (23-90)		63.9 (23-90)		65.0 (23-90)	

Abbreviations: MN, melanocytic naevi; PT, primary tumor; DM, distant metastasis.

Table S5 Description of tissue microarray specimens stratified into $p53^{neg}/p21^{low}$ and $p53^{pos}/p21^{high}$ cohorts.

Parameter	Cohort 1: $p53^{neg}/p21^{low}$		Cohort 2: $p53^{pos}/p21^{high}$	
	**TMAs characteristics**			
**No. of specimens**	*n*	%	*n*	%
	26	74.3	9	25.7
**No of patients**	*n*	%	*n*	%
	24	72.7	9	27.3
	**Clinicopathological Characteristics**			
**Sex**	*n*	%	*n*	%
Female	13	54.2	1	11.1
Male	11	45.8	8	88.9
**Age at Diagnosis**	Mean in years (range)			
	63.2 (31-84)		55.8 (28-75)	
**Specimen type**				
MN	5	19.2	-	-
PT	8	30.8	3	33.3
DM	13	50.0	6	66.6

Abbreviations: MN, melanocytic nevus; PT, primary tumor; DM, distant metastasis.

Supplemental video

Video S1 Ectopic expression of IER2 in mouse 3T3-IER2-H2B-GFP fibroblasts causes morphological changes and cell division defects. 3T3 cells with RSL-inducible Ier2 expression were stably transfected to express GFP-labeled histone H2B in their nuclei. The 3T3-Ier2-H2B-GFP cells were stimulated with either DMSO (left panel) or with RSL to induce Ier2 expression (right panel). The cells were time-lapse imaged for 48 hours, with one frame being taken every 5 minutes.

References

1. Hoek KS, Schlegel NC, Brafford P, Sucker A, Ugurel S, Kumar R et al. Metastatic potential of melanomas defined by specific gene expression profiles with no BRAF signature. *Pigment Cell Res* 2006; 19: 290-302.
2. Widmer DS, Cheng PF, Eichhoff OM, Belloni BC, Zipser MC, Schlegel NC et al. Systematic classification of melanoma cells by phenotype-specific gene expression mapping. *Pigment Cell Melanoma Res* 2012; 25: 343-353.
